# Trajectories of childhood social isolation in a nationally representative cohort: Associations with antecedents and early adulthood outcomes

**DOI:** 10.1002/jcv2.12073

**Published:** 2022-05-11

**Authors:** Katherine N. Thompson, Candice L. Odgers, Bridget T. Bryan, Andrea Danese, Barry J. Milne, Lily Strange, Timothy Matthews, Louise Arseneault

**Affiliations:** ^1^ Social, Genetic and Developmental Psychiatry Centre Institute of Psychiatry Psychology and Neuroscience King's College London London UK; ^2^ Social Science Research Institute Duke University Durham North Carolina USA; ^3^ Department of Psychological Science University of California Irvine Irvine California USA; ^4^ Department of Child & Adolescent Psychiatry Institute of Psychiatry Psychology & Neuroscience King's College London London UK; ^5^ National and Specialist CAMHS Trauma, Anxiety, and Depression Clinic South London and Maudsley NHS Foundation Trust London UK; ^6^ Faculty of Arts Centre of Methods and Policy Application in the Social Sciences University of Auckland Auckland New Zealand; ^7^ Faculty of Science Department of Statistics University of Auckland Auckland New Zealand

**Keywords:** developmental trajectories, growth mixture modelling, longitudinal research, mental health, social isolation

## Abstract

**Background:**

This study examined early life antecedents of childhood social isolation, whether these factors accounted for poor outcomes of isolated children, and how these associations varied according to patterns of stability and change in childhood isolation.

**Methods:**

Participants included 2232 children from the Environmental Risk (E‐Risk) Longitudinal Twin Study. We conducted growth mixture modelling (GMM) on combined parent and teacher reports of children's social isolation when children were 5, 7, 10 and 12 years, and we assessed associations with age‐5 antecedents and age‐18 outcomes using regression analyses.

**Results:**

We identified three linear developmental trajectories of increasing (4.75%), decreasing (5.25%) and low stable (90.00%) social isolation. Age‐5 attention deficit hyperactivity disorder (ADHD) symptoms, emotional problems, prosocial behaviours, maternal personality (openness) and size of school were associated with the decreasing trajectory of social isolation. When controlling for these antecedents, increasingly isolated children were still more likely to experience ADHD symptoms, loneliness, lower job optimism and lower physical activity at age 18.

**Conclusions:**

Isolated children follow distinct patterns of change over childhood and isolation seems most detrimental to health at the time it is experienced. Social isolation can be a valuable indicator of co‐occurring problems and provide targets for mental health intervention in young people.

## Graphical abstract

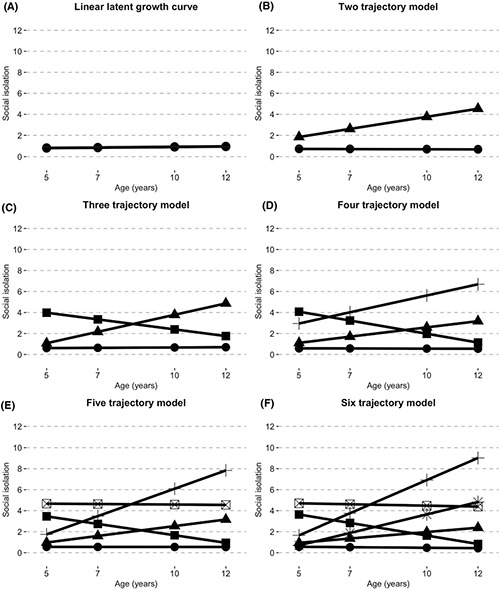

We identified three developmental trajectories of increasing, decreasing and low stable social isolation using the Environmental Risk Longitudinal Twin Study. Age‐5 child characteristics predicted childhood social isolation, and isolated children had worse outcomes at age 18. We showed that childhood social isolation can be an indicator of co‐occurring problems and provide avenues for mental health intervention in young people.

**Key points**

- Little is known about the developmental trajectories of social isolation in childhood, why some children become isolated and implications for later outcomes
- We show social isolation is neither common nor static; isolated children follow distinct patterns of change
- Children with ADHD, internalising, and lower prosocial behaviours were more likely than others to be isolated early in childhood
- Isolated children were, as adults, more likely to have ADHD symptoms, conduct disorder symptoms, loneliness, and lower career optimism
- Social isolation could be a valuable indicator of co‐occurring problems
- More longitudinal research is needed to assess bidirectional associations and timely intervention techniques that simultaneously reduce isolation and mental health problems to prevent deficits in health and functioning

## INTRODUCTION

Positive social relationships provide companionship, guidance and a source of support in times of stress (Cohen & Wills, 1985; Weiss, 1973). Social isolation occurs when there is a lack of these social relationships and interpersonal connections (Cacioppo et al., 2011) and can negatively impact physical and mental health throughout the lifespan (Holt‐Lunstad, 2017). Isolated adults are more likely to have symptoms of depression, accelerated cognitive decline and even premature mortality compared to those who are not isolated (Cacioppo & Cacioppo, 2014; Holt‐Lunstad et al., 2015; Santini et al., 2020). Social isolation can occur at any point in life and a growing body of evidence indicates that childhood isolation constitutes a major risk factor for poor health in adulthood (Caspi et al., 2006).

Social isolation in childhood could be experienced differently to that of adults. Rather than a loss of contact with other adults, childhood social isolation is dependent on belonging to a peer group. Childhood social isolation has been previously conceptualised as the extent to which children are socially connected to other children (Caspi et al., 2006; Danese et al., 2009; Lacey et al., 2014). Social isolation in childhood can be particularly detrimental as peer connections become increasingly important, cognitive processes rapidly develop and mental health problems become apparent and persistent (Orben et al., 2020). Children who experience social isolation or loneliness (the subjective feeling that relationships are inadequate) are more likely to have mental and physical health problems both concurrently and across time (Loades et al., 2020). Isolated children are at risk of depression, cardiovascular problems, inflammation, low educational attainment and obesity in adulthood (Caspi et al., 2006; Danese et al., 2009; Lacey et al., 2014). Moreover, persistent social isolation across developmental periods has been shown to have a cumulative impairing influence on health (Caspi et al., 2006; Loades et al., 2020; Yang et al., 2016).

It is unclear, however, whether social isolation in childhood follows a uniform pattern, or whether there are groups of children who follow different long‐term trends. By assessing the emergence and developmental pattern of social isolation in childhood, we can identify distinct courses that may be differently associated with poor outcomes. Social isolation is an experience that is influenced by the environment, thus can be dynamic. Previous work suggested that social isolation is transient in childhood (Matthews et al., 2015). Longitudinal studies can model developmental pathways by separating individuals into trajectory groups, represented by different starting points and patterns of change over time (Morneau‐Vaillancourt et al., 2021; van Dulmen & Goossens, 2013). Life course trajectories of social isolation identified groups of people that have never been isolated (71.6%), isolated in childhood only (14.3%), adulthood only (10.1%) and persistently isolated (4%; Lay‐Yee et al., 2021). Loneliness trajectory studies also show substantial variation between subgroups of children that represent transient experiences for some and prolonged for others (e.g., Qualter et al., 2013). Longitudinal trajectory studies can help in clarifying patterns of change in social isolation across childhood, elucidating why some children become socially isolated in the first instance and follow a particular trend and determining if persistent or enduring periods of childhood isolation are associated with poor outcomes later in life.

Childhood social isolation has been associated with earlier low self‐control, fearfulness, having a teen‐aged mother, maltreatment, a single‐parent family and change in residency, independent of developmental patterns (Lay‐Yee et al., 2021). These associations with child characteristics, the family environment and social factors extends to loneliness research; children who follow chronic persistent trajectories have been distinguished by internalising symptoms, aggression, negative temperament, passive play and family income, which in turn increased the risk for poor mental health outcomes (Qualter et al., 2013; Schinka et al., 2013). There could be patterns of social connection that have an impact over and above subjective appraisals of social connections (feeling lonely). Our study fills the gap in the literature on childhood specific trajectories of social isolation and how these patterns over time relate to poor health in early adulthood, whilst accounting for these relevant risk factors.

Using data from a UK nationally representative longitudinal cohort study, we aimed to (1) identify developmental trajectories of social isolation across ages 5, 7, 10 and 12; (2) test associations between these trajectories and age‐5 antecedents including societal factors, family home environment and child characteristics; and (3) explore associations between social isolation trajectories and age‐18 outcomes including mental health, physical health, coping and functioning, and employment prospects.

## METHODS

### Participants

Participants were members of the Environmental Risk (E‐Risk) Longitudinal Twin Study, which tracks the development of 2232 British children. The sample was drawn from a larger birth cohort of twins born in England and Wales in 1994–1995 (Trouton et al., 2002). Full details about the sample are reported elsewhere (Moffitt & the E‐Risk Study Team, 2002). Briefly, E‐Risk was constructed in 1999–2000, when 1116 families (93% of those eligible) with same‐sex 5‐year‐old twins participated in home‐visit assessments. This sample comprised 56% monozygotic and 44% dizygotic twin pairs; sex was evenly distributed within zygosity (49% male); 90% of participants were of White ethnicity. The sample represents socioeconomic conditions in the UK, as reflected in the families' distribution on neighbourhood‐level socioeconomic indices (Odgers et al., 2012; Reuben et al., 2020). Follow‐up home visits were conducted when the children were aged 7 (98% participation), 10 (96%), 12 (96%) and 18 years (93%; see Appendix S1). Visits at ages 5–12 included assessments with participants and their mother (primary caretaker) and at age 18 included interviews with participants. The Joint South London and Maudsley and the Institute of Psychiatry Research Ethics Committee approved each phase of the study. Parents gave informed consent and participants gave assent between 5 and 12 years, then informed consent at age 18.

### Measures

#### Social isolation

At ages 5, 7, 10 and 12, social isolation was assessed using six items from the children's behaviour checklist (CBCL; Achenbach, 1991a) and the matching items from the teacher's report form (TRF; Achenbach, 1991b). We used Caspi et al.’s (2006) approach whereby childhood social isolation is conceptualised as social rejection or withdrawal. Two items mirror those used by Caspi et al. (2006): ‘would rather be alone than with others’ and ‘not liked by other children[pupils]’. As seen in Matthews et al. (2015) four additional items were used to capture isolation due to rejection and limited interaction with other children: ‘does not get along with other children[pupils]’, ‘feels or complains that no‐one loves him/her’, ‘withdrawn, does not get involved with others’ and ‘complains of loneliness’. Responses were scored 0 (not true), 1 (somewhat true) and 2 (often true). Mothers completed the questionnaire in a face‐to‐face interview at each age, and teachers responded to the same items by post. Items were summed to create mother and teacher scales at each age (*r*'s at each age = 0.26–0.31). These correlations are consistent with previous parent and teacher correlations for children's behaviour and are likely accounted for by situational specificity (Achenbach et al., 1987). Thus, we averaged mothers' and teachers' reports to form a combined score which integrates observations from different settings. Cronbach's alpha (α) for the combined score at each age ranged from 0.68 to 0.78 (α for mother report at each age = 0.64–0.76, α for teacher report at each age = 0.68–0.76). A total of 2079 (93.14%) children had social isolation data at all time points. Missingness was handled using maximum likelihood estimation, thus all individuals were included in analyses (*N* = 2232; see Appendix S1).

#### Childhood antecedents

We assessed age‐5 antecedents from five domains: social factors, home environment, parent characteristics, child neurodevelopment, and child emotional development. Social factors captured urban residence (ACORN neighbourhood classification), neighbourhood vandalism, problems with neighbours, number of children attending the child's school, the number of children at school eligible for free school meals, and family socioeconomic status (SES). Home environment variables included mothers not living with the biological fathers since birth, number of siblings, domestic violence, child harm, maternal social support, number of stimulating activities involving mother and child, and maternal warmth. Parent characteristics comprised measures of maternal depression, maternal personality, parental antisocial behaviour, and alcoholism. Child neurodevelopment variables captured intelligence quotient (IQ), executive functioning, and theory of mind. Child emotional and behavioural development variables comprised externalising behaviours, internalising problems, hyperactivity and impulsivity behaviours (attention deficit hyperactivity disorder [ADHD]), and prosocial behaviours. The CBCL items used to construct the social isolation measure were removed when deriving the internalising problems scale. Full details are provided in Appendix S2.

#### Early adulthood outcomes

We measured age‐18 outcomes which cover domains of mental health, physical health, coping and functioning, and employment prospects. Mental health measures included past year diagnoses and symptom scales of major depression, generalised anxiety, ADHD, conduct disorder, alcohol and cannabis dependence, experiences of psychosis, and instances of self‐harm or suicide attempts between ages 12 and 18. Participants also reported whether they had seen a professional for mental health problems in the past year. The physical health domain comprised measures of body mass index (BMI), C‐reactive protein (CRP; a marker of inflammation), day‐to‐day physical activity, and daily smoking. The coping and functioning domain included measures of loneliness, life satisfaction, coping with stress, problematic technology use and poor sleep quality (low Pittsburgh sleep quality index score). To capture employment prospects, participants were asked about their highest qualification level, if they were currently in employment/education, job search behaviour, career optimism, and perceived job preparedness. Variable selection and description were adapted from Matthews et al. (2019); full details in Appendix S2.

### Statistical analysis

#### Trajectories of childhood social isolation

We used growth mixture modelling (GMM) to estimate trajectories of social isolation at ages 5, 7, 10 and 12. GMM identifies latent classes of individuals who follow similar trajectories that differ from one overall trend (Jung & Wickrama, 2008). First, we fitted latent growth curve models (LGCM) to determine the overall population trajectory form of social isolation (linear, quadratic and log‐linear) and if participants significantly varied from this trajectory. Second, we followed recommendations to conduct latent class growth analysis (LCGA) as an initial modelling step before specifying GMM (Jung & Wickrama, 2008; Muthén & Muthén, 2000; van de Schoot et al., 2017). LCGA is a less computationally demanding model that performs a version of GMM that specifies zero variance within a class (Nagin & Odgers, 2010). This can often be unrealistic and as all variance is explained through the defined classes, LCGA is known to overestimate the number of classes present (Herle et al., 2020). Third, we computed GMM for two to six latent classes with age terms from our best‐fitting LGCM form. This number of classes was informed by past studies of social isolation and loneliness trajectories, which suggest four to six classes (e.g., Eccles et al., 2020; Lay‐Yee et al., 2021). The best fitting model was identified using standard fit statistics and class size considerations (≥5% of sample), whilst giving preference to the most parsimonious model that is developmentally meaningful (van de Schoot et al., 2017). Model fit was indicated by Akaike information criterion (AIC; lower preferable), sample size adjusted Bayesian information criterion (aBIC; lower preferable), Vuong‐Lo‐Mendell‐Rubin likelihood ratio test (VLMR‐LRT; *p* < 0.05 indicates better fit than model with one fewer class) and entropy (classification accuracy; higher is preferable). We examined the quality of the best fitting model using the probability of correct class membership (>0.8 considered accurate). Clustering was specified to account for the non‐independent observations of same‐sex twins. Results are reported in accordance with GRoLTS Checklist (van de Schoot et al., 2017).

#### Associations between social isolation trajectories with childhood antecedents and outcomes in early adulthood

We used the standard three‐step method (van de Schoot et al., 2017) to assess how social isolation trajectory groups were associated with age‐5 antecedents and age‐18 outcomes. For the antecedents, we fitted univariate multinomial regressions for all variables separately and then added them all into a single multivariate multinomial regression, controlling for sex. The trajectory class that indicates the expected normative trend of low social isolation over time and has the highest *N* will be used as the reference group. For the associations with the outcomes, we fitted linear regression models for continuous variables and logistic regression models for binary variables, controlling for sex and SES. We adjusted standard errors to account for the non‐independence of twin observations (Williams, 2000). As a sensitivity check, analyses were repeated using the three‐step approach with adjustment for classification errors and multiple‐testing corrections were applied for univariate models. We repeated all outcome models whilst controlling for significant antecedents.

Analyses were conducted in Mplus(v8), R(v4.0.3) and STATA(v16). The analysis plan was pre‐registered (https://sites.google.com/site/moffittcaspiprojects/home/concept‐paper_2020/thompsonk_2020) and code is on GitHub (https://knthompson26.github.io/Childhood‐social‐isolation‐trajectories_GMM/).

## RESULTS

### Developmental trajectories of childhood social isolation

**FIGURE 1:**
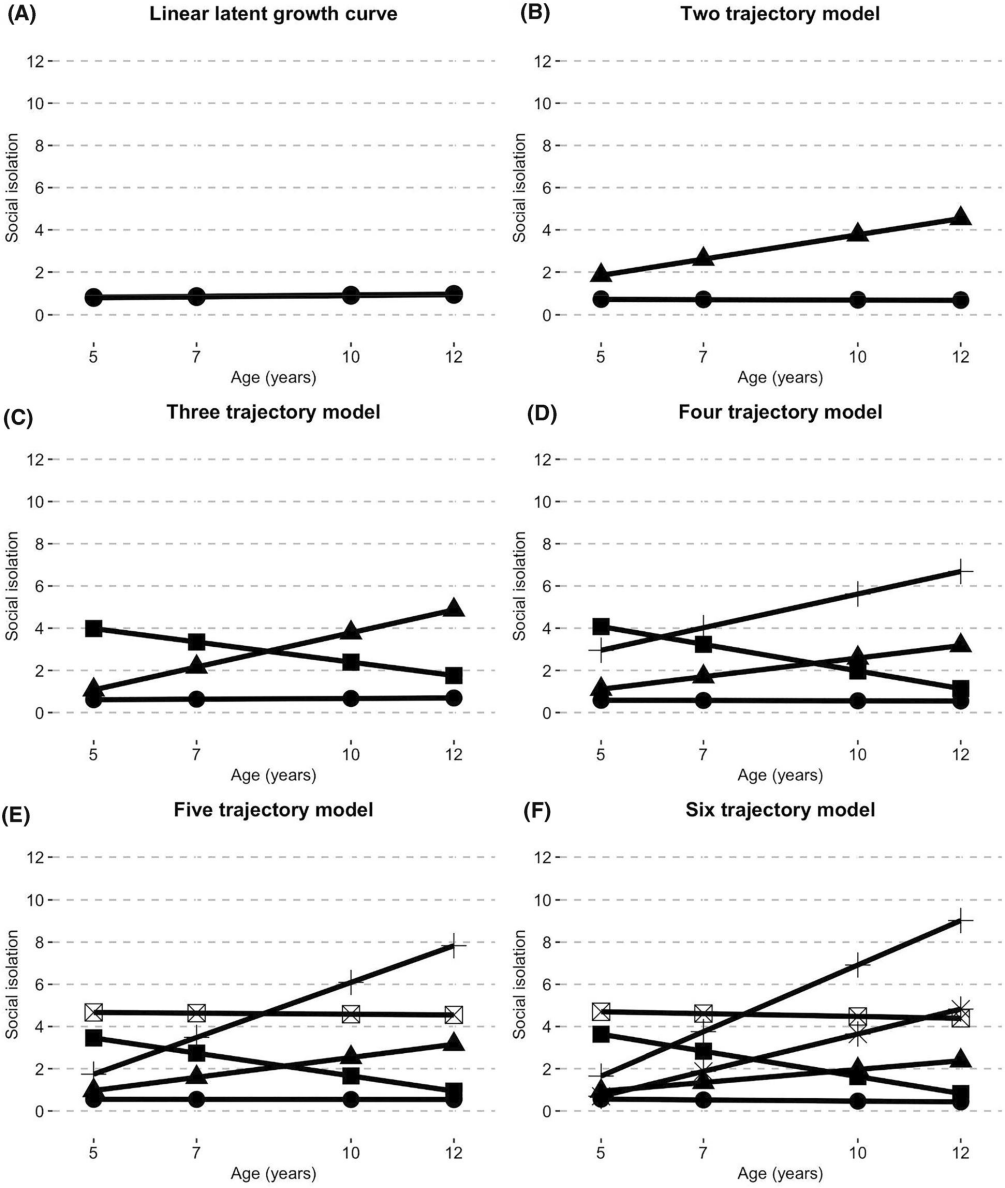
Trajectories of childhood social isolation from ages 5 to 12. Panel A shows a linear latent growth curve model. Panels B through F show linear two to six class growth mixture models.

Linear LGCM best fit the data and showed significant variance in initial levels of isolation (intercept:0.72, *p* < 0.001) and change over time (slope:0.02, *p* < 0.001; see Appendix S3), which supported further examination of distinct trajectories. LCGA suggested four trajectories as a preliminary estimation (see Appendix S4), but this method can overestimate the number of classes (Herle et al., 2020). Using GMM, we selected three distinct developmental trajectories of social isolation as the best solution (Figure 1; Table 1). These social isolation trajectories represent a *low stable* group (*N* = 2009; 90.00%), an *increasing* group (*N* = 106; 4.75%) and a *decreasing* group (*N* = 117; 5.25%). All model statistics are provided in Appendices S5 and S6.

**TABLE 1:** Growth mixture model fit indices for two through six class solutions

Number of classes	AIC	aBIC	Entropy	VLMR‐LRT	Sample percentage of smallest group
2	25,447.50	25,477.90	0.951	0.0344	7
**3**	**24,922.10**	**24,960.11**	**0.952**	**0.0587**	**5**
4	24,629.97	24,675.57	0.936	0.8906	2
5	24,352.62	24,400.76	0.942	0.0541	1
6	24,163.34	24,219.08	0.928	0.2071	1

*Note*: Probability of correct classification was high (>0.87) for all groups. Model information, sample descriptives, average trajectory isolation scores and quadratic GMM provided in Appendix S5 and S6. Best fitting model indicated in bold.

Abbreviations: AIC, Akaike's information criterion; aBIC, sample size adjusted Bayesian information criterion; VLMR‐LRT, Vuong‐Lo‐Mendell‐Rubin likelihood ratio test.

### Associations between childhood antecedents and social isolation trajectories

**FIGURE 2:**
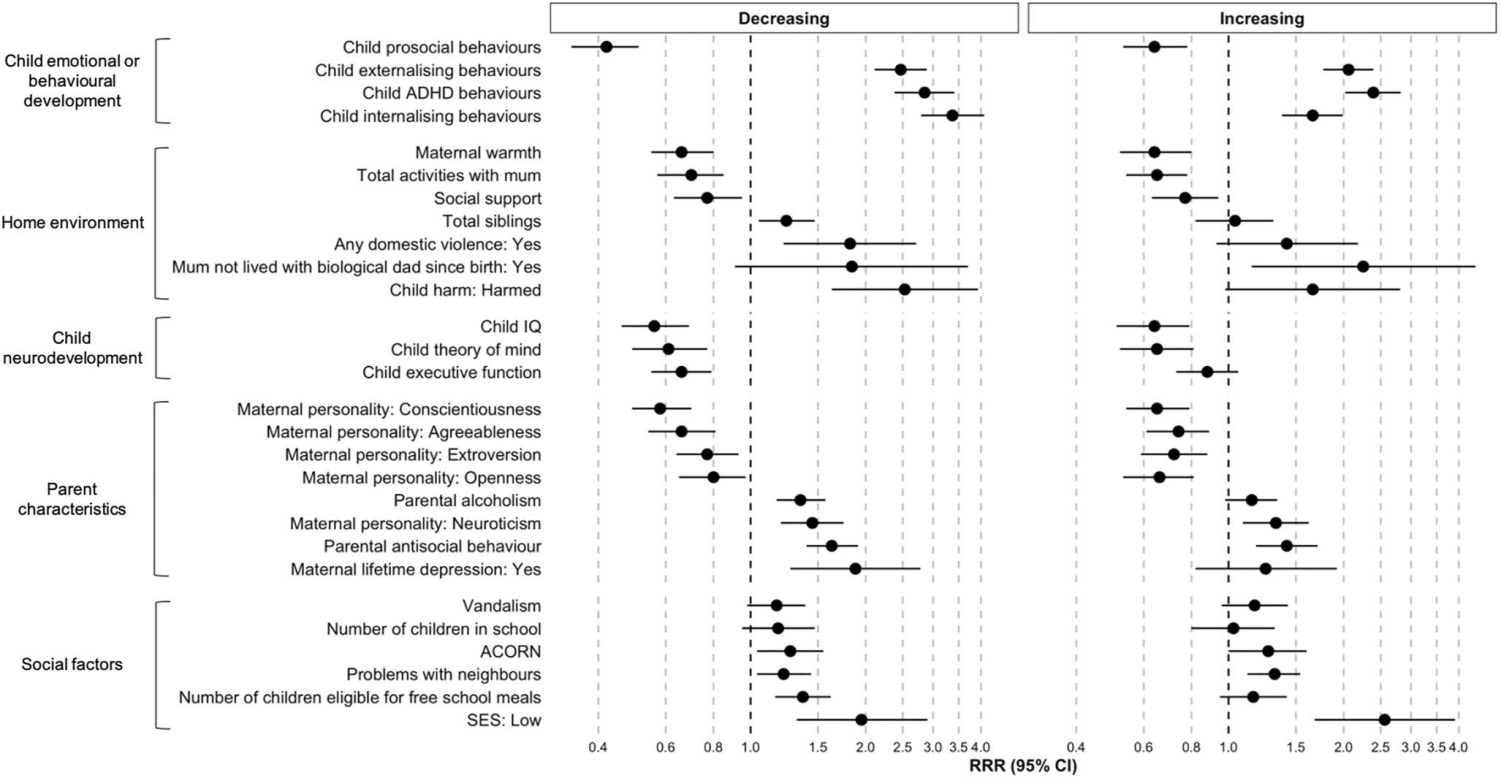
Univariate associations between increasing and decreasing social isolation trajectories with age‐5 antecedents (*N* total = 2232, *N* increasing = 106, *N* decreasing = 117). RRR and 95% CI are shown for all associations. The low stable trajectory group was the reference group and all regressions controlled for sex.

Age‐5 antecedents were individually associated with increasing and decreasing social isolation trajectories from ages 5 to 12 (Figure 2; Full relative risk ratios [RRR] and confidence intervals [CI] are in Appendix S7). Children with increased ADHD behaviours and emotional problems, and those from low SES families had the highest odds of being isolated (either increasing or decreasing). Children with increased prosocial behaviours, high IQ, and those with warm mothers were least likely to be isolated. When all antecedents were simultaneously entered in the regression model, children with increased ADHD behaviours had increased odds of following the increasing (RRR = 1.90, 95%CI = 1.40, 2.57) or decreasing (RRR = 1.78, 95%CI = 1.25, 2.54) trajectory. Children with increased emotional problems (RRR = 3.02, 95%CI = 2.29, 3.97), a mother with higher personality trait of openness (RRR = 1.67, 95%CI = 1.12, 2.50), and who were attending a bigger school (RRR = 1.43, 95%CI = 1.07, 1.93) had an increased likelihood of following the decreasing trajectory, but not the increasing trajectory. Children with more prosocial behaviours (RRR = 0.59, 95%CI = 0.43, 0.81) were less likely to follow the decreasing trajectory. In other words, they were less likely to be concurrently isolated than those in the low stable group. Children with higher executive functioning had a marginal increased likelihood of following the increasing trajectory (RRR = 1.30, 95%CI = 1.02, 1.66). The low stable trajectory was the reference group in all regression analyses. Multiple testing corrections did not influence conclusions, thus original estimates are reported (see Appendix S7).

### Associations between social isolation trajectories and outcomes in early adulthood

**FIGURE 3:**
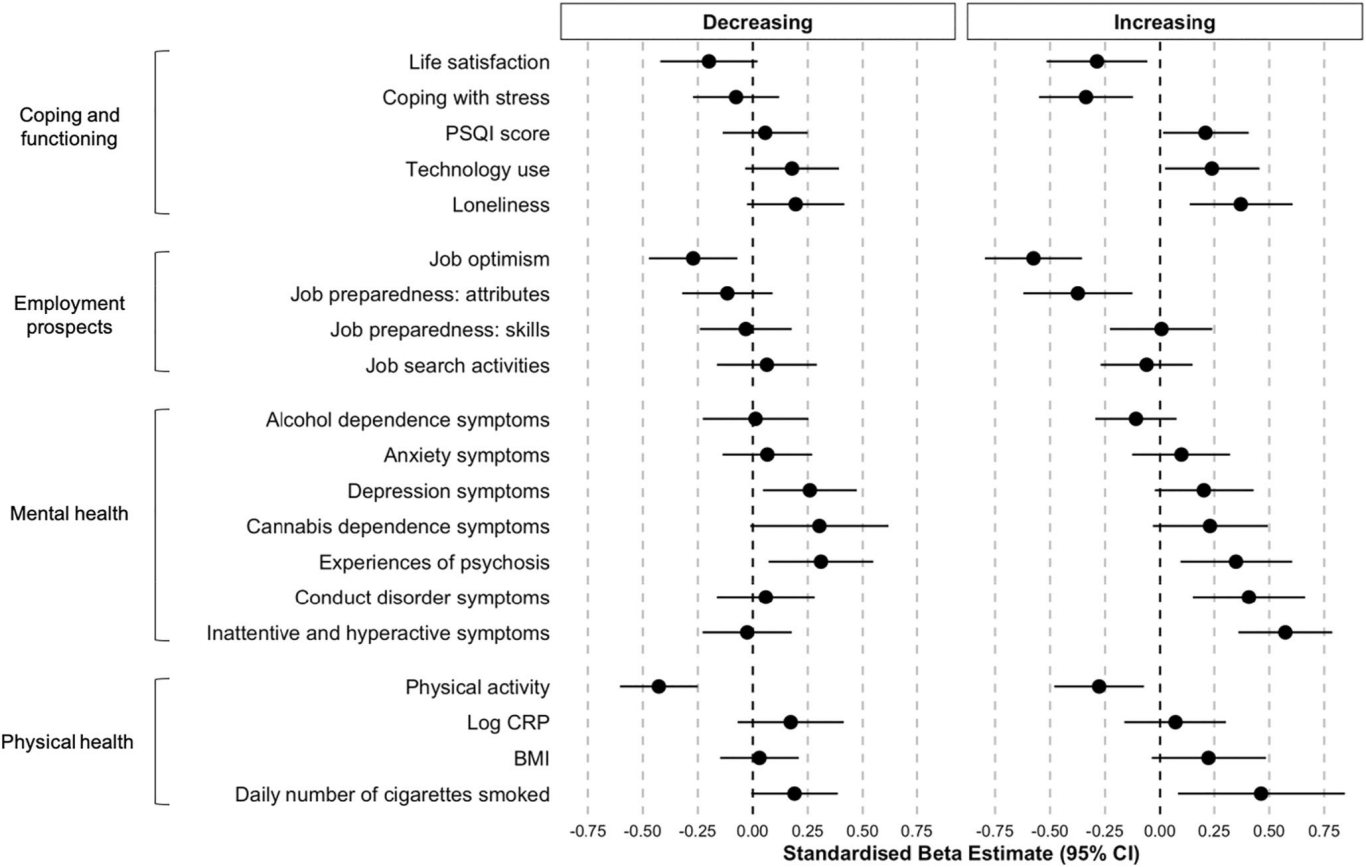
Univariate associations between increasing and decreasing social isolation trajectories and age‐18 continuous outcomes (*N* total = 2232, *N* increasing = 106, *N* decreasing = 117). Beta (β) estimates and 95% confidence intervals are shown for all associations.

Increasing and decreasing trajectories of social isolation from age 5 to 12 were associated with poor outcomes at age 18 (Figure 3). Children who had higher levels of isolation than the low stable group at any point in childhood (followed the increasing or decreasing trajectory) were more likely to report psychotic‐like experiences, less likely to be optimistic about their career and undertook less daily physical activity. For binary outcomes, they were more likely to have attempted suicide (increasing odds ratio [OR] = 1.99, 95%CI = 1.16, 3.39; decreasing OR = 1.69, 95%CI = 1.02, 2.79) and be dependent on cannabis (increasing OR = 2.22, 95%CI = 1.08, 4.54; decreasing OR = 2.11, 95%CI = 1.01, 4.39). Children who followed the increasing trajectory exclusively showed poorer coping and functioning at age 18. Compared to the low‐stable isolated group, they were also more likely to have a diagnosis of ADHD (OR = 2.15, 95%CI = 1.15, 4.01) and conduct disorder (OR = 1.91, 95%CI = 1.16, 3.15), report overall higher levels of ADHD and conduct disorder symptoms, as well as greater risk of using mental health services (OR = 1.91, 95%CI = 1.10, 3.32). Moreover, they were more likely to be out of employment and education (OR = 2.07, 95%CI = 1.24, 3.47), to believe they had fewer ‘soft skills’ to offer a potential employer, and smoke cigarettes daily at age 18. Children who followed the decreasing trajectory exclusively were more likely to have increased depression symptoms and a marginal significant risk for a depression diagnosis (OR = 1.57, 95%CI = 1.00, 2.45, *p* = 0.05). Full OR/β, CI and multiple testing corrections presented in Appendix S8.

We further tested the associations between social isolation trajectories and outcomes at age 18 whilst controlling for significant antecedents. Associations with age‐18 ADHD symptoms, conduct disorder symptoms, loneliness, job optimism and physical activity remained significant. Associations with other outcomes became non‐significant, indicating that they were accounted for by pre‐existing factors. Children who followed the increasing isolation trajectory remained more likely to have greater ADHD symptoms at age 18 (*β* = 0.35, *p* = 0.003), greater conduct disorder symptoms (*β* = 0.30, *p* = 0.031), to feel lonely (*β* = 0.29, *p* = 0.018) and to be less optimistic about their career prospects (*β* = −0.40, *p* = 0.001). Children who followed the decreasing isolation trajectory were less likely to take part in regular physical activity at age 18 (*β* = −0.27, *p* = 0.012), with a similar, but non‐significant, trend for children in the increasing trajectory (*β* = −0.21, *p* = 0.064). OR/β and CI, sex interactions and posterior probability sensitivity analyses are presented in Appendices S9–S11. All results were replicated using the three‐step approach with adjustment for classification errors (see Appendix S12).

## DISCUSSION

We identified three developmental trajectories of increasing, decreasing and low stable social isolation across childhood. Our findings show that social isolation is neither common nor static; isolated children follow distinct patterns of change over childhood. Children with certain characteristics were more likely than others to be socially isolated early in childhood. Compared to those who were not isolated, isolated children were also more likely to have difficulties with mental health and daily functioning as they became young adults. While several associations were accounted for by pre‐existing characteristics, increasing childhood social isolation remained independently associated with ADHD symptoms, conduct disorder symptoms, loneliness, and less career optimism in early adulthood. We demonstrate that childhood social isolation can have concurrent and sometimes bidirectional detrimental influences on health.

Our study provides new insight into the developmental pattern of social isolation in a large UK nationally representative cohort. Based on repeated measures from 5 to 12 years, our findings showed that most children were not isolated, and for those who were, they were not consistently isolated across childhood. We add explanatory value over and above the use of average levels and linear growth curves of social isolation through identifying groups of children that have distinct patterns of isolation that peak at different developmental time points. The increasing and decreasing patterns of severity and stability are similar to those observed by Lay‐Yee et al. (2021), although we did not observe a chronic‐stable trajectory. This is not unexpected, as their chronic‐stable social isolation trajectory emerged in adulthood. Substantial patterns of change have also been reported in the loneliness literature at comparable ages (e.g., Eccles et al., 2020). The mid‐to‐late childhood period is marked with life transitions, such as progression through school, which could reflect changes in peer relations. Chronic social isolation may become more stable later in adolescence at a time where individuals become autonomous, peer relationships increase in complexity and social cognition develops exponentially (Andrews et al., 2021).

Childhood social isolation is a known risk factor for poor health in later life (e.g., Bennett et al., 2020). We showed that when establishing temporal precedence, the association between poor mental health and social isolation appears bidirectional. For example, we found children with ADHD behaviours were likely to be isolated concurrently (decreasing isolation) and at age 18 (increasing isolation). Children with ADHD are more likely than their peers to exhibit impulsive social behaviours (Gardner & Gerdes, 2015). Other children could hold negative attributions about children with ADHD and respond with rejection or victimisation (McQuade et al., 2018), isolating children with ADHD from their peers. We also found that increasing childhood social isolation was associated with age‐18 ADHD symptoms, over and above prior symptoms. Isolated children have limited opportunities to learn social skills, norms and communication that come from interacting with others and receiving social feedback (Gardner & Gerdes, 2015). As these social skill deficits are also apparent in children with ADHD (Ros & Graziano, 2018), isolation could exacerbate or expose ADHD behaviours as children get older. Our findings highlight the complex associations between social isolation and mental health and emphasise the importance of recognising social isolation in children as a valuable indicator of co‐occurring problems.

Previous research noted the cumulative impact of chronic social isolation, whereby isolation at multiple developmental points has a dose–response negative influence on health (Caspi et al., 2006). We showed that age‐5 antecedents were primarily associated with the decreasing trajectory of social isolation, and the increasing trajectory with age‐18 outcomes. There are several interpretations for these findings. First, associations could be prominent between measures that are assessed closely in time. Second, the detrimental cumulative effect of social isolation may only be applicable when isolation arises later in childhood. This idea supports previous findings, where individuals with late‐onset ADHD, but not those with early‐onset, reported being isolated at age 18 (Agnew‐Blais et al., 2018). Third, children could be concurrently isolated at the time they are experiencing other problems. Rather than a cumulative effect, severity at the time of adversity plays a key role in mental health. We showed that age‐5 antecedents predicted concurrent (decreasing trajectory), rather than long‐term (increasing trajectory), isolation. For example, age‐5 prosocial behaviours reduced the likelihood of concurrent isolation, potentially through creating beneficial peer relationships and buffering the association between social withdrawal and peer exclusion (Freitas et al., 2019). But in contrast to previous findings (Ma et al., 2020a), this reduction did not continue to age 18. We conclude that social isolation at any point can be detrimental to children, and it may take an extended period for the cumulative effect of adversity to impact health. This challenges the added value of conceptualising social isolation as developmental trajectories and perhaps it is more useful for future research to consider the time frame where isolation is most severe.

Some limitations merit acknowledgement. First, 2‐year gaps in the assessment of social isolation may have missed important information on change within the childhood period. Utilising more frequent measurement throughout childhood and adolescence might provide a more detailed pattern of change. Second, our findings from a twin sample may not generalise to populations of singletons as all participants had at least one sibling of the same age. However, twins can become isolated from their peers despite and due to their relationship with their co‐twin. For example, twins that spend lots of time with their co‐twin could be less likely to develop relationships outside the dyad. Third, trajectories derived by GMM are statistically modelled and are not directly measured by participant reports. Caution is warranted when interpreting and using these groupings as a proxy for directly observed entities (Peugh & Fan, 2012). Fourth, the items used to measure childhood social isolation were not originally designed to assess social isolation and were derived from another validated measure assessing overall child behaviour (CBCL). Research in adult populations often uses a singular item to report loss or lack of social contact (e.g., loss of a spouse or living alone). This approach cannot be applied when assessing social isolation in childhood. If social contact is limited to that of only parents and siblings, children can be seen as isolated and do not get the full breadth of social experiences required for development. Therefore, we use age‐appropriate items from previous work (Matthews et al., 2015) which builds upon Caspi et al.’s (2006) approach that child social isolation is conceptualised as social rejection or withdrawal. Fifth, trajectory patterns found here are commonly identified when using GMM to capture sample variation. This can even occur in the same sample when separating out participants based on age or duration of observation periods, providing contradictory conclusions of the trajectories present (Sher et al., 2011). That said, we replicated trajectories previously identified in a separate cohort of adults (Lay‐Yee et al., 2021).

Our findings suggest several avenues to inform clinical interventions and future research. Social isolation changes throughout childhood and young isolated children could transition through this experience without intervention. However, we show that isolated children would benefit from support at the time they are isolated to reduce the risk of long‐lasting problems. There is value in increasing social support as an intervention target with young people, as a dense and meaningful social network has been shown to be protective against outcomes such as suicide ideation and attempt (Calati et al., 2019). However, interventions for social isolation using community mentorship (King et al., 2018), school‐based approaches (Griffin et al., 2017) and social skills training (Storebø et al., 2019) report limited success, small effects or mixed conclusions (Loades et al., 2020; Ma et al., 2020b). For example, social skills training for children with ADHD has been minimally effective as ADHD social difficulties reflect inconsistent interactions rather than lack of social knowledge (Aduen et al., 2018). Considering potential bidirectional associations, isolated children should be offered interventions to increase social interaction in combination with those that address mental health problems. More longitudinal research is needed to track the complexity and development of the bidirectional associations between social isolation and mental health across the lifespan.

## CONFLICT OF INTEREST

Louise Arseneault is a member of the Editorial Advisory Board for JCPP *Advances*. The remaining authors have declared that they have no competing or potential conflicts of interest.

## ETHICAL CONSIDERATIONS

The Joint South London and Maudsley and the Institute of Psychiatry Research Ethics Committee approved each phase of the study. Parents gave informed consent and participants gave assent between 5 and 12 years, then informed consent at age 18.

## AUTHOR CONTRIBUTIONS

**Katherine Thompson:** Conceptualization; data curation; formal analysis; methodology; visualization; writing – original drafts; writing – review & editing. **Candice Odgers:** Formal analysis; methodology; writing – review & editing. **Bridget T Bryan:** Writing – review & editing. **Andrea Danese:** Writing – review & editing. **Barry Milne:** Writing – review & editing. **Lily Strange:** Writing – review & editing. **Timothy Matthews:** Conceptualization; Methodology; Supervision; Writing – review & editing. **Louise Arseneault:** Conceptualization; Funding acquisition; Methodology; Project administration; Resources; Supervision; Writing – review & editing.

### OPEN RESEARCH BADGES

This article has earned an Open Materials badge for making publicly available the components of the research methodology needed to reproduce the reported procedure and analysis. All materials are available at http://knthompson26.github.io/Childhood‐social‐isolation‐trajectories_GMM/.

## Supporting information

Supporting Information S1

## Data Availability

The dataset reported in the current article is not publicly available due to lack of informed consent and ethical approval, but is available on request by qualified scientists. Requests require a concept paper describing the purpose of data access, ethical approval at the applicant's institution and provision for secure data access. We offer secure access on the King's College campus. All data analysis scripts and results files are available for review.
