# Supplementary material for: Trajectories of childhood social isolation in a nationally representative cohort: Associations with antecedents and early adulthood outcomes
Source: JCPP Adv. 2022 May 11;2(2):e12073. doi: 10.1002/jcv2.12073 (PMC10242821; doi:10.1002/jcv2.12073)
Supplement: Supplementary file 1 — Supporting Information S1 [file JCV2-2-e12073-s001.docx]

Trajectories of childhood social isolation in a nationally representative cohort: Associations with antecedents and early adulthood outcomes

Supporting Information

**Katherine N. Thompson**^1^, **Candice L. Odgers**^2,3^, **Bridget T. Bryan**^1^, **Andrea Danese**^1,4,5^, **Barry Milne**^6,7^**, Lily Strange**^1^, **Timothy Matthews***^1^, and **Louise Arseneault***^1^

^1^ Social, Genetic and Developmental Psychiatry Centre, Institute of Psychiatry, Psychology and Neuroscience, King's College London, London, UK

^2^Social Science Research Institute, Duke University, Durham, NC, USA

^3^Department of Psychological Science, University of California Irvine, CA, USA

^4^ Department of Child & Adolescent Psychiatry, Institute of Psychiatry, Psychology & Neuroscience, King's College London, London, UK

^5^ National and Specialist CAMHS Trauma, Anxiety, and Depression Clinic, South London and Maudsley NHS Foundation Trust, London, UK

^6^Centre of Methods and Policy Application in the Social Sciences, Faculty of Arts, University of Auckland, New Zealand

^7^Department of Statistics, Faculty of Science, University of Auckland, New Zealand

*Joint senior author

Table of Contents

Appendix [S1. Sample characteristics 4](#_Toc100322854)

[Missingness at age 18 4](#_Toc100322855)

[Missing social isolation data in E-Risk 4](#_Toc100322856)

[Skewness and kurtosis 4](#_Toc100322857)

Appendix [S2. Overview of antecedent and outcome variables 5](#_Toc100322858)

Appendix [S3. Latent growth curve models (LCGM) 7](#_Toc100322859)

[Fit indices for all LCGM 7](#_Toc100322860)

[Means and variances for LCGM 7](#_Toc100322861)

Appendix [S4. Latent Class Growth Analysis (LCGA) 8](#_Toc100322862)

[Fit statistics 8](#_Toc100322863)

[Posterior probability statistics for 4 class LCGA 8](#_Toc100322864)

[Figure 1. LCGA mean trajectories for two through to six classes 9](#_Toc100322865)

Appendix [S5. Statistics for the GMM three-class model 9](#_Toc100322866)

[Trajectory social isolation means for the 3-class model 10](#_Toc100322867)

[Figure 2. Individual and estimated mean three class trajectories 10](#_Toc100322868)

[Sample descriptives stratified by class 11](#_Toc100322869)

[Means and variances 11](#_Toc100322870)

Appendix [S6. Growth mixture models (GMM) with a quadratic term 12](#_Toc100322871)

[Key findings 12](#_Toc100322872)

[Means and variances 12](#_Toc100322873)

[Figure 3: Two class GMM with quadratic term 13](#_Toc100322874)

[Change in classification from linear and quadratic models 14](#_Toc100322875)

Appendix [S7. Association with childhood antecedents 15](#_Toc100322876)

[Figure 4. Antecedent multicollinearity 15](#_Toc100322877)

[Relative Risk Ratios (RRR) and 95% confidence intervals (CI) 16](#_Toc100322878)

[A. Table 15. Univariate models (class ~ variable) 16](#_Toc100322879)

[B. Table 16. Multivariate model (class ~ variable1 + variable2 + variable3) 18](#_Toc100322880)

[Multiple testing (FDR) correction 19](#_Toc100322881)

Appendix [S8. Associations with outcomes at age 18 20](#_Toc100322882)

[Figure 5. Outcome variables multicollinearity 20](#_Toc100322883)

[20](#_Toc100322884)

[Table 18. Outcome logistic regression models with Odds Ratio (OR) estimates and 95% confidence intervals (binary.variable ~ class) 21](#_Toc100322885)

[Table 19. Outcome linear regression models with Beta estimates and 95% confidence intervals (linear.variable ~ class) 21](#_Toc100322886)

[Multiple testing (FDR) correction 23](#_Toc100322887)

[Logistic regression models 23](#_Toc100322888)

[Linear regression models 23](#_Toc100322889)

Appendix [S9. Association with outcomes at age 18 whilst controlling for antecedents at age 5 23](#_Toc100322890)

[Table 20. Logistic outcome models, controlling for antecedents (logistic.variable ~ class + antecedent1 + antecedent2 + antecedent3) 23](#_Toc100322891)

[Table 21. Linear outcome models, controlling for antecedents (linear.variable ~ class + antecedent1 + antecedent2 + antecedent3) 24](#_Toc100322892)

Appendix [S10. Sex interactions 25](#_Toc100322893)

[Not in employment or education 25](#_Toc100322894)

[Service use 25](#_Toc100322895)

[Cannabis dependence 26](#_Toc100322896)

[BMI 26](#_Toc100322897)

[CRP 26](#_Toc100322898)

[PSQI 26](#_Toc100322899)

Appendix [S11. Posterior probability sensitivity analysis 27](#_Toc100322900)

[Antecedents 27](#_Toc100322901)

[1. Univariate models 27](#_Toc100322902)

[2. Multivariate model 29](#_Toc100322903)

[Outcomes 32](#_Toc100322904)

[1. Logistic 32](#_Toc100322905)

[2. Linear 33](#_Toc100322906)

[Outcomes controlling for antecedents 35](#_Toc100322907)

[1. Logistic 35](#_Toc100322908)

[2. Linear 36](#_Toc100322909)

Appendix [S12. 3-Step sensitivity analysis 38](#_Toc100322910)

[Antecedents: Manual 3-step approach for predictors in Mplus 38](#_Toc100322911)

[Increasing class regressions (Increasing class ~ variable1 + variable2 …) 39](#_Toc100322912)

[Decreasing class regressions (Decreasing class ~ variable1 + variable2 …) 39](#_Toc100322913)

[Outcomes: Automatic 3-Step BCH method sensitivity analysis in Mplus 40](#_Toc100322914)

[References 43](#_Toc100322915)

# Appendix S1. Sample characteristics

##

## Missingness at age 18

There were no differences between those who did and did not take part at age 18 in terms of socioeconomic status (SES) assessed when the cohort was initially defined, $\chi2$(2, N=2,232)=0.86, *p*=0.65; age-5 IQ scores, t(2,208)=0.98, p=0.33; or age-5 emotional or behavioural problems, t(2,230)=0.40, *p*=0.69 and t(2,230)=0.41, *p*=0.68, respectively.

## Missing social isolation data in E-Risk

Cronbach alpha for the combined score was 0.68 at age 5, 0.73 at age 7, 0.75 at age 10 and 0.78 at age 12. Missingness was 0% at age 5, 2.42% at age 7, 4.21% at age 10 and 3.90% at age 12. At the individual level, 3.99% (N=89) of children were missing one assessment, 2.06% (N=46) of children were missing two and 0.81% (N=18) of children were missing the remaining three after the initial age 5 assessment. Social isolation data was assumed to be missing at random (MAR) as there were no significant differences between those who were missing data from three assessments and those who were not on IQ (t=1.22, *p*=0.23), internalising behaviours (t=-0.56, *p*=0.58) and externalising behaviours (t=0.36, *p*=0.72). There were also no differences in social isolation at age five (t=0.71, *p*=0.49) for individuals missing the remaining three time points and those who were not. Thus, all individuals were included in subsequent analyses (N=2,232).

## Skewness and kurtosis

| **Table 1**. Mplus calculated skewness and kurtosis statistics for three-class GMM model | | | | | |
| --- | --- | --- | --- | --- | --- |
|  |  | **Sample value** | **Mean** | **SD** | **p-value** |
| **Kurtosis** | **Three class model** | 76.947 | 30.457 | 1.131 | <0.001 |
|  | Age 5 | 12.471 | 2.644 | 0.642 | <0.001 |
|  | Age 7 | 9.063 | 0.862 | 0.335 | <0.001 |
|  | Age 10 | 7.638 | 0.805 | 0.294 | <0.001 |
|  | Age 12 | 8.902 | 2.564 | 0.597 | <0.001 |
| **Skew** | **Three class model** | 25.227 | 3.825 | 0.504 | <0.001 |
|  | Age 5 | 2.2759 | 1.123 | 0.137 | <0.001 |
|  | Age 7 | 2.526 | 0.563 | 0.097 | <0.001 |
|  | Age 10 | 2.328 | 0.556 | 0.093 | <0.001 |
|  | Age 12 | 2.510 | 1.121 | 0.131 | <0.001 |

#

#

#

#

# Appendix S2. Overview of antecedent and outcome variables

| **Table 2.** Measures of age-5 antecedents and age-18 outcome variables | | | |
| --- | --- | --- | --- |
|  | **Domain** | **Measure** | **Description** |
| *Antecedents at age 5* | | | |
|  | **Social factors** | Urban residence:  Neighbourhood classification | ACORN Neighbourhood classification based on 2001 CENSUS (Odgers, Caspi, Bates, et al., 2012; Odgers, Caspi, Russell, et al., 2012) |
|  |  | Neighbourhood:  Vandalism;  Problems with neighbours | Vandalism: graffiti and damage to property, cars broken into or stolen.  Problems with neighbours: noisy neighbours, arguments, loud parties. |
|  |  | School:  Total number of children attending the school;  Total number of children eligible for free school meals | The Office for Standards in Education, Children's Services and Skills (OFSTED) report from school. |
|  |  | Family:  Socioeconomic status (SES) | Tertile of standardised composite of parental income, education, and occupation. |
|  | **Home environment** | Family structure | Mother has not lived with the biological father since the birth of the twins; Number of siblings. |
|  |  | Exposure to domestic violence | Exposure to any domestic violence in the first 5 years since participants’ birth measured by Conflict Tactics Scales (Straus, 1979) |
|  |  | Physical maltreatment or child harm | Multi-Site Child Development Project standardised protocol, developed by (Jaffee et al., 2004) |
|  |  | Maternal social support | Composite of time taken to reach closest family members and number of close friends in the immediate area. |
|  |  | Stimulating activities with parent | Composite of stimulating activities. For example, been to the park, been to the cinema, been on a long walk. |
|  |  | Maternal warmth | Maternal expressed emotion scales based on the five minute speech sample method. (Caspi et al., 2004) |
|  | **Parent characteristics** | Maternal depression | Lifetime history of a major depressive episode based on DSM-IV criteria (American Psychiatric Association, 1994) |
|  |  | Maternal personality | Big five inventory (John, 1999) |
|  |  | Parental antisocial behaviour and aggression | Lifetime presence of symptoms of conduct disorder or antisocial personality disorder in either parent, based on DSM-IV criteria (American Psychiatric Association, 1994) |
|  |  | Parental alcoholism | A Shortened Version of the Michigan Alcoholism Screening Test (Pokorny et al., 1972) |
|  | **Child neurodevelopment** | Intelligence Quotient (IQ) | Assessed using a short form of the Wechsler Preschool and Primary Scale of Intelligence-Revised (Wechsler, 1990) |
|  |  | Executive functioning | Composite of three tests: Mazes (WPPSI subtest), nonverbal analogue of the Stroop task, and Sentence working memory. (Gerstadt et al., 1994; Grodzinsky & Diamond, 1992). |
|  |  | Theory of mind | Battery of Theory of Mind tasks (Hughes et al., 2000) |
|  | **Child emotional or behavioural development** | Externalising behaviours: antisocial behaviour, aggression, and delinquency subscales | CBCL/TRF. (Achenbach & Edelbrock, 1991) |
|  |  | Internalising behaviours: anxiety, withdrawn, and somatic subscales | CBCL/TRF. (Achenbach & Edelbrock, 1991) |
|  |  | Hyperactivity/impulsivity: Attention-Deficit Hyperactivity Disorder items, including inattentive, impulsive, and hyperactive symptoms | CBCL/TRF. (Achenbach & Edelbrock, 1991) |
|  |  | Prosocial behaviours | CBCL/TRF. (Achenbach & Edelbrock, 1991) |
| *Early adulthood Outcomes at age 18* | | | |
|  | **Mental health** | Mental health diagnosis: MDD, GAD, ADHD, conduct disorder, PTSD, alcohol dependence or cannabis dependence | Past-year diagnosis according to DSM-IV or DSM-V criteria, assessed via structured clinical interview. (American Psychiatric Association, 1994) |
|  |  | Experience of psychosis | Items from the Structured Interview for Psychosis-Risk Syndromes (SIPS) |
|  |  | Self-harm or suicide attempt | Reports of at least one instance of self-harm or suicide attempt between ages 12 and 18 |
|  |  | Service use | Any visit to a general practitioner, psychiatrist or counsellor/psychotherapist for mental health problems in the past year |
|  | **Physical health** | Body Mass Index (BMI) | Calculated from height and weight measurements taken by interviewers at the home visit |
|  |  | C-reactive protein (CRP) | Collected via dried blood spots. mg/l values were log-transformed prior to analysis |
|  |  | Physical activity | Daily physical activity during work/college or leisure time, measured using the Stanford Brief Activity Survey |
|  |  | Daily smoking | Number of cigarettes smoked per day |
|  | **Coping and functioning** | Loneliness | UCLA Loneliness Scale, Version 3 |
|  |  | Life satisfaction | Global life satisfaction measured via the Satisfaction with Life Scale |
|  |  | Coping with stress | Count of strategies used when experiencing stress in relation to finances, relationships, college or work. Four positively-coded items (For example, ‘talk with other people about it’, ‘take steps to solve the problem’) and four negatively-coded items (‘withdraw or spend more time alone’, ‘obsess about problems’) were combined, with higher scores reflecting more positive coping strategies. |
|  |  | Problematic technology use | Compulsive use of digital technology such as the internet, email, social networking, mobile phones and text messaging. Measured using an adapted version of the Compulsive Internet Use Scale |
|  |  | Sleep | The Pittsburgh sleep quality index |
|  | **Employment prospects** | Not in employment, education or training (NEET) | Participants report whether they were currently employed or studying |
|  |  | Low qualifications | Self-rating of professional and technical skills. For example, writing and computer programming. Self-rating of ‘soft’ skills. For example, communication and teamwork |
|  |  | Optimism | Self-rated perceptions of participants’ ability to get ahead in their careers |
|  |  | Job search activities | Total number of job-seeking activities participants have undertaken. For example, applied for a job or looked at job vacancies pages. |

# Appendix S3. Latent growth curve models (LCGM)

## Fit indices for all LCGM

We identified the overall population trajectory through fitting linear and quadratic latent growth curve models (LCGM) for unadjusted and log-transformed social isolation scores, with age as the metric of time. Mean social isolation scores appeared consistent across ages 5 (0.81, SD=1.13), 7 (0.83, SD=1.18), 10 (0.94, SD=1.30) and 12 (0.94, SD=1.37) years. LGCM for linear, quadratic, log-linear and log-quadratic terms showed similar fit (**Table 3**). Although the log-adjusted models had lower aBIC (reduction of ~15,000); to aid model interpretation the most parsimonious linear growth curve was selected as the best fit and used in subsequent analyses. The linear LCGM had a significant intercept (0.81, *p*<0.001) and slope (0.02, *p*<0.001), thus on average participants scored 0.81 at age five with slight increase over time. It also showed significant variance in the intercept (0.72, *p*<0.001) and slope (0.02, *p*<0.001), which justified the examination of distinct social isolation trajectories in our sample (**Table 4**).

| **Table 3.** Fit statistics for linear and quadratic latent growth curve models for unadjusted and log transformed scores | | | | | | | |
| --- | --- | --- | --- | --- | --- | --- | --- |
| **Model** | **Parameters** | **AIC** | **BIC** | **aBIC** | **CFI** | **TLI** | **RMSEA** |
| **Linear growth model** | **9** | **26268.18** | **26319.58** | **26290.98** | **0.991** | **0.989** | **0.027** |
| Log linear growth model | 9 | 11069.13 | 11120.53 | 11091.93 | 0.993 | 0.992 | 0.030 |
| Quadratic growth model | 13 | 26255.23 | 26329.47 | 26288.16 | 0.997 | 0.984 | 0.033 |
| Log quadratic growth model | 13 | 11064.84 | 11139.08 | 11097.77 | 0.997 | 0.984 | 0.043 |
| AIC: Akaike’s Information Criterion; BIC: Bayesian Information Criterion; aBIC: sample size adjusted Bayesian Information Criterion; CFI: comparative fit index; TLI: Tucker-Lewis Index; RMSEA: root mean square error of approximation. Best fitting model indicated in bold. | | | | | | | |

## Means and variances for LCGM

##

| **Table 4.** Means and variances for linear latent growth curve | | | | |
| --- | --- | --- | --- | --- |
|  | **Parameter** | **Estimate** | **Standard error** | ***p* value** |
| **Means** | I | 0.808 | 0.026 | <0.001 |
|  | S | 0.021 | 0.005 | <0.001 |
| **Variances** | I | 0.718 | 0.079 | <0.001 |
|  | S | 0.020 | 0.003 | <0.001 |

| **Table 5.** Means and variances for quadratic latent growth curve | | | | |
| --- | --- | --- | --- | --- |
|  | **Parameter** | **Estimate** | **Standard error** | ***p* value** |
| **Means** | I | 0.806 | 0.027 | <0.001 |
|  | S | 0.025 | 0.015 | 0.093 |
|  | Q | -0.001 | 0.002 | 0.773 |
| **Variances** | I | 0.788 | 0.127 | <0.001 |
|  | S | 0.090 | 0.029 | 0.002 |
|  | Q | 0.001 | <0.001 | 0.002 |

# Appendix S4. Latent Class Growth Analysis (LCGA)

LCGA specifies models similar to GMM but assumes all individuals within a class share exactly the same trajectory (no within-class individual variation), which can make theoretical interpretation difficult (Herle et al., 2020; van der Nest et al., 2020). LCGA of two through six classes showed the four-class solution as the best fit according to goodness of fit criteria (**Table 6**). The four class model showed substantial decrease in aBIC, compared to the three-class model (aBIC decrease=546.26), with classes that represent a *low stable* group, *low increasing* group, *high increasing* group, and *decreasing* group. Although the VLMR-LRT test was nonsignificant (*p*=0.80), the addition of the fourth class captures more variation in trajectory membership without decreasing sample proportions (2% for three and four class models).

## Fit statistics

| **Table 6.** Fit statistics for latent class growth analysis (LCGA) for 2 through to 6 classes | | | | | | |
| --- | --- | --- | --- | --- | --- | --- |
| **Model** | **AIC** | **BIC** | **aBIC** | **Entropy** | **VLMR LRT** | **Class Percentages** |
| 2 | 26053.35 | 26104.75 | 26076.15 | 0.945 | 0.0008 | 10, 90 |
| 3 | 25279.97 | 25348.50 | 25310.37 | 0.920 | 0.0608 | 15, 2, 82 |
| **4** | **24726.10** | **24811.76** | **24764.11** | **0.932** | **0.8002** | **81, 5, 11, 2** |
| 5 | 24440.05 | 24542.84 | 24485.65 | 0.934 | 0.0728 | 1, 2, 80, 10, 7 |
| 6 | 24242.80 | 24362.72 | 24296.01 | 0.924 | 0.2323 | 3, 1, 5, 2, 76, 14 |
| AIC: Akaike’s Information Criterion; BIC: Bayesian Information Criterion; aBIC: sample size adjusted Bayesian Information Criterion; VLMR-LRT: Vuong-Lo-Mendell-Rubin Likelihood Ratio Test. Best fitting and chosen model indicated in bold. | | | | | | |

##

## Posterior probability statistics for 4 class LCGA

The probability of correct classification was high (>0.836) for all groups identified by the LCGA.

| **Table 7**. Probability of correct classification per class | |
| --- | --- |
| Class | Classification probabilities |
| Low stable | 0.987 |
| Decreasing | 0.860 |
| Low increasing | 0.836 |
| High increasing | 0.956 |

##

##

## Figure 1. LCGA mean trajectories for two through to six classes

Latent classes of combined parent and teacher reported childhood social isolation from 5 to 12 years of age. Panels A through E show latent class growth curves for two to six classes. The solid lines represent mean class trajectories estimated by each model, with the relevant key beneath each panel. The slope of the LCGA trajectories was modelled using linear terms for all models. All models were clustered for family ID.


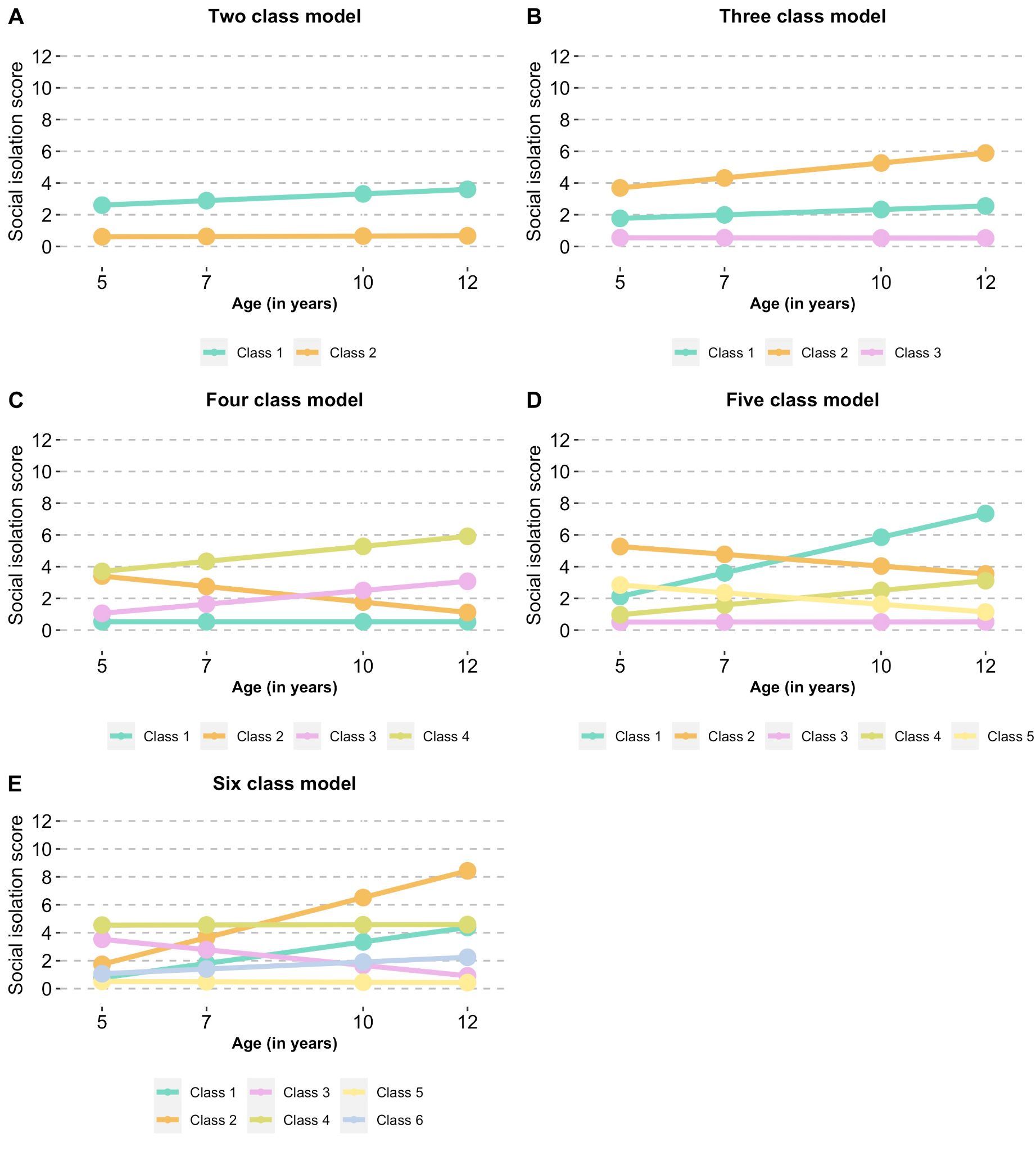


##

# Appendix S5. Statistics for the GMM three-class model

Five and six class model solutions reported negative slope variance, thus, for these models slope variance was manually fixed to zero in a partially constrained GMM. The three and four class models were replicated using seed values from the two highest log-likelihood to check the global solution was obtained (Wickrama et al., 2016). The probability of correct classification was high for the low stable (0.99), increasing (0.87), and decreasing (0.90) trajectory groups. Residual variances were estimated freely as is the default setting in Mplus.

##

## Trajectory social isolation means for the 3-class model

| **Table 8**. Social isolation means at each time point stratified by class | | | | |
| --- | --- | --- | --- | --- |
| **Class** | **Mean at age 5** | **Mean at age 7** | **Mean at age 10** | **Mean at age 12** |
| Low stable | 0.605 | 0.632 | 0.718 | 0.673 |
| Increasing | 1.291 | 1.848 | 3.545 | 5.027 |
| Decreasing | 3.984 | 3.374 | 2.347 | 1.771 |

## Figure 2. Individual and estimated mean three class trajectories

Observed individual trajectories (N=2,232) of the three identified classes of social isolation overlayed by the estimated mean trajectories of each class. Low stable trajectories are indicated in blue, increasing trajectories in orange, and decreasing trajectories in pink.


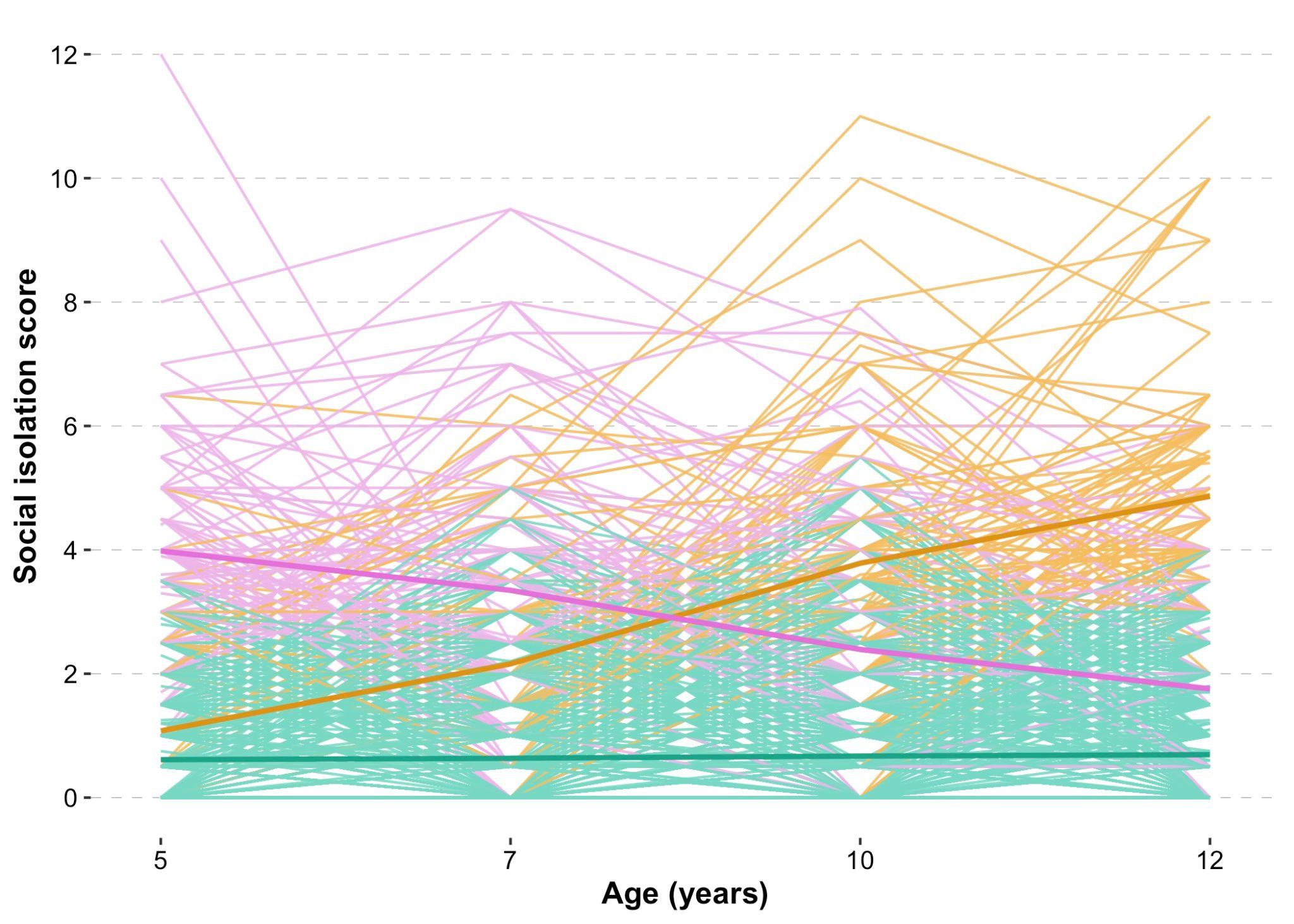


## Sample descriptives stratified by class

| **Table 9**. Sample descriptives of sex, zygosity, socioeconomic status, and ethnicity stratified by social isolation classes | | | | | | | | | |
| --- | --- | --- | --- | --- | --- | --- | --- | --- | --- |
|  |  | **Low stable** | | **Increasing** | | **Decreasing** | | **Total** | |
|  |  | **N** | **%** | **N** | **%** | **N** | **%** | **N** | **%** |
| **Sex** | **Male** | 961 | 47.83 | 62 | 58.49 | 69 | 58.97 | 1092 | 48.92 |
|  | **Female** | 1048 | 52.17 | 44 | 41.51 | 48 | 41.03 | 1140 | 51.08 |
| **Zygosity** | **MZ** | 1133 | 56.40 | 55 | 51.89 | 54 | 46.15 | 1242 | 55.65 |
|  | **DZ** | 876 | 43.60 | 51 | 48.11 | 63 | 53.85 | 990 | 44.35 |
| **SES** | **Low** | 630 | 31.36 | 57 | 53.77 | 55 | 47.01 | 742 | 33.24 |
|  | **Middle** | 681 | 33.90 | 27 | 25.47 | 30 | 25.64 | 738 | 33.06 |
|  | **High** | 698 | 34.74 | 22 | 20.75 | 32 | 27.35 | 752 | 33.69 |
| **Ethnicity** | **White** | 1818 | 90.49 | 102 | 96.23 | 98 | 83.76 | 2018 | 90.41 |
|  | **Asian** | 78 | 3.88 | 2 | 1.89 | 10 | 8.55 | 90 | 4.03 |
|  | **Black** | 36 | 1.79 | 0 | 0.00 | 6 | 5.13 | 42 | 1.88 |
|  | **Mixed race** | 7 | 0.35 | 0 | 0.00 | 1 | 0.85 | 8 | 0.36 |
|  | **Other** | 70 | 3.48 | 2 | 1.89 | 2 | 1.71 | 74 | 3.32 |
| SES: socioeconomic status; DZ: dizygotic twins; MZ: monozygotic twins. | | | | | | | | | |

## Means and variances

| **Table 10.** Means and variances for three class linear growth mixture model of social isolation | | | | | |
| --- | --- | --- | --- | --- | --- |
|  |  | **Parameter** | **Estimate** | **Standard error** | ***p* value** |
| *Class 1* | **Means** | I | 0.612 | 0.031 | 0.000 |
|  |  | S | 0.012 | 0.006 | 0.045 |
|  | **Variances** | I | 0.171 | 0.055 | 0.002 |
|  |  | S | 0.003 | 0.002 | 0.125 |
| *Class 2* | **Means** | I | 1.077 | 0.202 | 0.000 |
|  |  | S | 0.541 | 0.062 | 0.000 |
|  | **Variances** | I | 0.171 | 0.055 | 0.002 |
|  |  | S | 0.003 | 0.002 | 0.125 |
| *Class 3* | **Means** | I | 3.983 | 0.369 | 0.000 |
|  |  | S | -0.318 | 0.053 | 0.000 |
|  | **Variances** | I | 0.171 | 0.055 | 0.002 |
|  |  | S | 0.003 | 0.002 | 0.125 |
| I: Intercept; S: Slope. | | | | | |

#

# Appendix S6. Growth mixture models (GMM) with a quadratic term

## Key findings

We re-computed the 2-6 class models with a quadratic growth term. The two and three class models converged with significant quadratic term means and variances (**Table 10**). However, the 4-6 class models had negative slope variance. When the slope variance was constrained to zero, the 4-6 class models resulted in non-convergence.

| **Table 11.** Fit statistics for two and three class quadratic growth mixture models of social isolation | | | | | | |
| --- | --- | --- | --- | --- | --- | --- |
| **Model** | **AIC** | **BIC** | **aBIC** | **Entropy** | **VLMR LRT p-value** | **Class Percentages** |
| Two Class | 25392.55 | 25489.63 | 25435.62 | 0.950 | 0.0562 | 93, 7 |
| Three Class | 24762.68 | 24882.60 | 24815.88 | 0.956 | 0.2802 | 90, 5, 5 |

##

## Means and variances

###

| **Table 12.** Means and variances for two class quadratic growth mixture models of social isolation | | | | | |
| --- | --- | --- | --- | --- | --- |
| *Class* 1 |  | **Parameter** | **Estimate** | **Standard error** | ***p* value** |
|  | **Means** | I | 0.714 | 0.030 | 0.000 |
|  |  | S | 0.033 | 0.022 | 0.127 |
|  |  | Q | -0.006 | 0.003 | 0.044 |
|  | **Variances** | I | 0.642 | 0.119 | 0.000 |
|  |  | S | 0.081 | 0.029 | 0.006 |
|  |  | Q | 0.001 | 0.001 | 0.013 |
| *Class 2* | **Means** | I | 1.979 | 0.227 | 0.000 |
|  |  | S | -0.087 | 0.254 | 0.732 |
|  |  | Q | 0.067 | 0.039 | 0.085 |
|  | **Variances** | I | 0.642 | 0.119 | 0.000 |
|  |  | S | 0.081 | 0.029 | 0.006 |
|  |  | Q | 0.001 | 0.001 | 0.013 |
| I: Intercept; S: Slope; Q: Quadratic slope. | | | | | |

| **Table 13**. Means and variances for three class quadratic growth mixture models of social isolation | | | | | |
| --- | --- | --- | --- | --- | --- |
| *Class* 1 |  | **Parameter** | **Estimate** | **Standard error** | ***p* value** |
|  | **Means** | I | 0.594 | 0.041 | 0.000 |
|  |  | S | 0.078 | 0.023 | 0.001 |
|  |  | Q | -0.010 | 0.003 | 0.001 |
|  | **Variances** | I | 0.244 | 0.095 | 0.010 |
|  |  | S | 0.073 | 0.28 | 0.010 |
|  |  | Q | 0.001 | 0.000 | 0.014 |
| *Class 2* | **Means** | I | 1.417 | 0.223 | 0.000 |
|  |  | S | -0.142 | 0.207 | 0.493 |
|  |  | Q | 0.095 | 0.031 | 0.002 |
|  | **Variances** | I | 0.244 | 0.095 | 0.010 |
|  |  | S | 0.073 | 0.28 | 0.010 |
|  |  | Q | 0.001 | 0.000 | 0.014 |
| *Class 3* | **Means** | I | 4.274 | 0.638 | 0.000 |
|  |  | S | -0.826 | 0.202 | 0.000 |
|  |  | Q | 0.069 | 0.026 | 0.008 |
|  | **Variances** | I | 0.244 | 0.095 | 0.010 |
|  |  | S | 0.073 | 0.28 | 0.010 |
|  |  | Q | 0.001 | 0.000 | 0.014 |
| I: Intercept; S: Slope; Q: Quadratic slope. | | | | | |

## Figure 3: Two class GMM with quadratic term


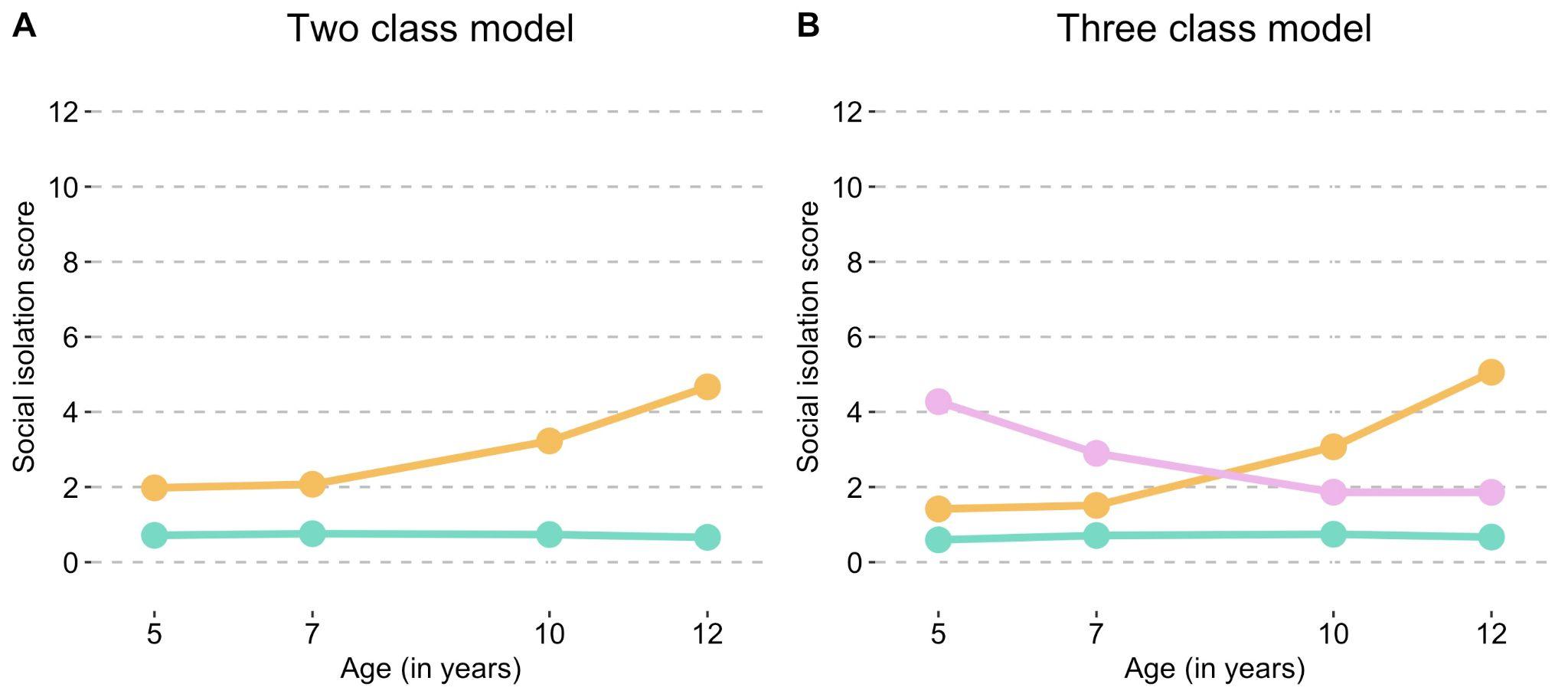


##

##

##

##

##

##

## Change in classification from linear and quadratic models

| **Table 14.** Participant direction of movement from linear to quadratic growth mixture models | |
| --- | --- |
| **Direction of movement** | **N** |
| Linear decreasing to quadratic increasing | 1 |
| Linear decreasing to the quadratic low stable | 28 |
| Linear increasing to the quadratic low stable | 19 |
| Linear low stable to quadratic decreasing | 11 |
| Linear low stable to quadratic increasing | 19 |

| **Table 15**. Class N for linear and quadratic growth mixture models | | | |
| --- | --- | --- | --- |
|  | **Increasing N** | **Decreasing N** | **Low stable N** |
| **Linear** | 106 | 117 | 2009 |
| **Quadratic** | 107 | 99 | 2026 |
| **Matching classification for linear and quadratic** | 87 | 88 | 1979 |

# Appendix S7. Association with childhood antecedents

## Figure 4. Antecedent multicollinearity

No antecedent variables breached the multicollinearity threshold (r>0.9).


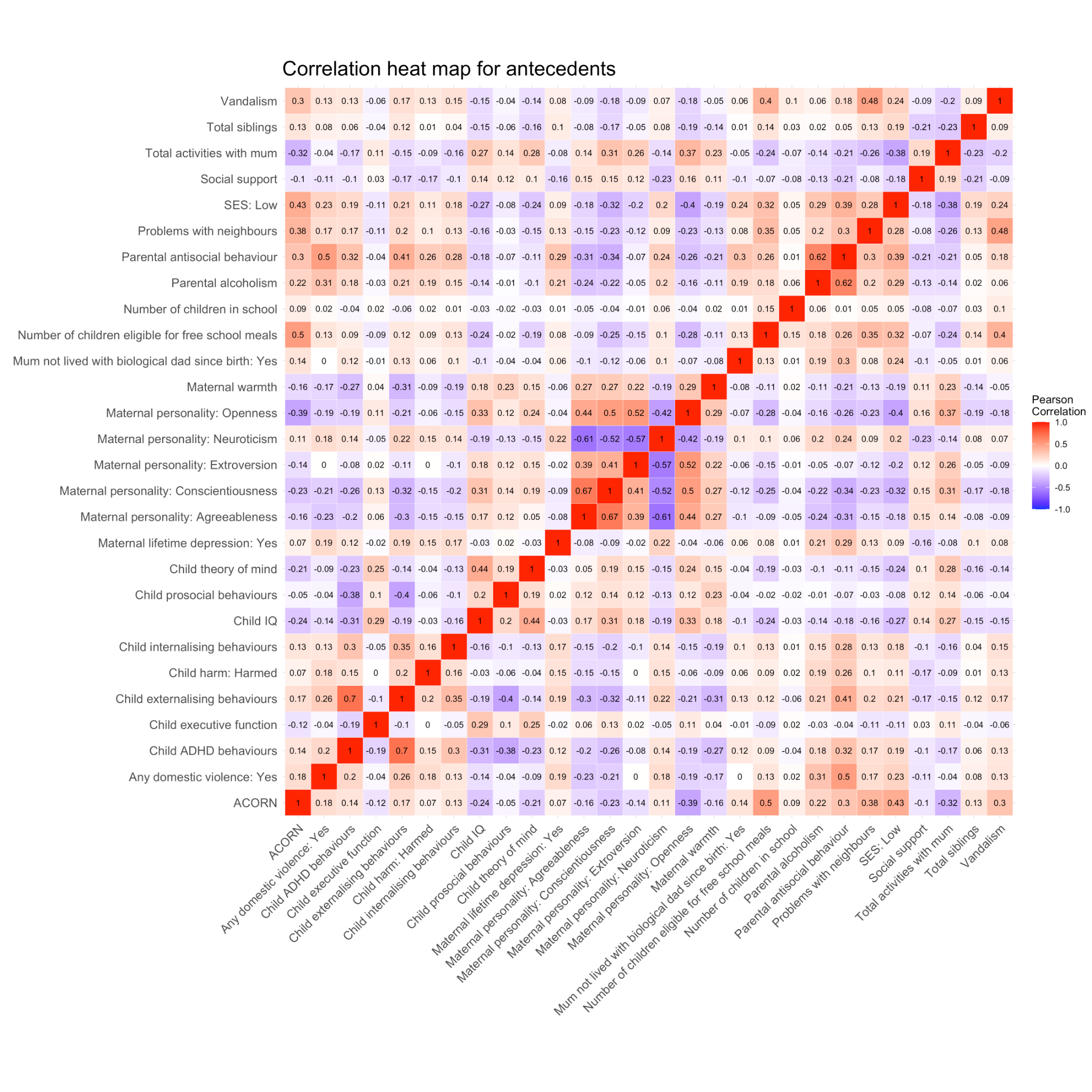


## Relative Risk Ratios (RRR) and 95% confidence intervals (CI)

### **Table 15**. Univariate models (class ~ variable)

| **Table 16.** Relative Risk Ratios (RRR) and 95% confidence intervals for univariate antecedent multinomial regression models | | | | | | |
| --- | --- | --- | --- | --- | --- | --- |
| **Class/**  **trajectory** | **Variable** | **RRR** | **95% CI low** | **95% CI high** | **p value** | **Domain** |
| Decreasing | SES: Low | 1.95 | 1.32 | 2.90 | **0.00** | Social domain |
| Decreasing | ACORN | 1.27 | 1.04 | 1.55 | **0.02** | Social domain |
| Decreasing | Vandalism | 1.17 | 0.98 | 1.39 | 0.08 | Social domain |
| Decreasing | Problems with neighbours | 1.22 | 1.04 | 1.44 | **0.01** | Social domain |
| Decreasing | Number of children in school | 1.18 | 0.95 | 1.47 | 0.14 | Social domain |
| Decreasing | Number of children eligible for free school meals | 1.37 | 1.16 | 1.62 | **0.00** | Social domain |
| Increasing | SES: Low | 2.56 | 1.68 | 3.91 | **0.00** | Social domain |
| Increasing | ACORN | 1.27 | 1.00 | 1.60 | **0.05** | Social domain |
| Increasing | Vandalism | 1.17 | 0.96 | 1.43 | 0.13 | Social domain |
| Increasing | Problems with neighbours | 1.32 | 1.12 | 1.54 | **0.00** | Social domain |
| Increasing | Number of children in school | 1.03 | 0.80 | 1.32 | 0.84 | Social domain |
| Increasing | Number of children eligible for free school meals | 1.16 | 0.95 | 1.42 | 0.14 | Social domain |
| Decreasing | Child harm: Harmed | 2.53 | 1.63 | 3.93 | **0.00** | Home domain |
| Decreasing | Total siblings | 1.24 | 1.05 | 1.47 | **0.01** | Home domain |
| Decreasing | Social support | 0.77 | 0.63 | 0.95 | **0.01** | Home domain |
| Decreasing | Total activities with mum | 0.70 | 0.57 | 0.85 | **0.00** | Home domain |
| Decreasing | Mum not lived with biological dad since birth: Yes | 1.84 | 0.91 | 3.70 | 0.09 | Home domain |
| Decreasing | Any domestic violence: Yes | 1.82 | 1.22 | 2.71 | **0.00** | Home domain |
| Decreasing | Maternal warmth | 0.66 | 0.55 | 0.80 | **0.00** | Home domain |
| Increasing | Child harm: Harmed | 1.66 | 0.98 | 2.81 | 0.06 | Home domain |
| Increasing | Total siblings | 1.04 | 0.82 | 1.31 | 0.75 | Home domain |
| Increasing | Social support | 0.77 | 0.63 | 0.94 | **0.01** | Home domain |
| Increasing | Total activities with mum | 0.65 | 0.54 | 0.78 | **0.00** | Home domain |
| Increasing | Mum not lived with biological dad since birth: Yes | 2.25 | 1.15 | 4.42 | **0.02** | Home domain |
| Increasing | Any domestic violence: Yes | 1.42 | 0.93 | 2.18 | 0.10 | Home domain |
| Increasing | Maternal warmth | 0.64 | 0.52 | 0.80 | **0.00** | Home domain |
| Decreasing | Maternal lifetime depression: Yes | 1.88 | 1.27 | 2.78 | **0.00** | Parent domain |
| Decreasing | Maternal personality: Openness | 0.80 | 0.65 | 0.97 | **0.03** | Parent domain |
| Decreasing | Maternal personality: Conscientiousness | 0.58 | 0.49 | 0.70 | **0.00** | Parent domain |
| Decreasing | Maternal personality: Extroversion | 0.77 | 0.64 | 0.93 | **0.01** | Parent domain |
| Decreasing | Maternal personality: Agreeableness | 0.66 | 0.54 | 0.81 | **0.00** | Parent domain |
| Decreasing | Maternal personality: Neuroticism | 1.45 | 1.20 | 1.75 | **0.00** | Parent domain |
| Decreasing | Parental antisocial behaviour | 1.63 | 1.40 | 1.91 | **0.00** | Parent domain |
| Decreasing | Parental alcoholism | 1.35 | 1.17 | 1.57 | **0.00** | Parent domain |
| Increasing | Maternal lifetime depression: Yes | 1.25 | 0.82 | 1.92 | 0.30 | Parent domain |
| Increasing | Maternal personality: Openness | 0.66 | 0.53 | 0.81 | **0.00** | Parent domain |
| Increasing | Maternal personality: Conscientiousness | 0.65 | 0.54 | 0.79 | **0.00** | Parent domain |
| Increasing | Maternal personality: Extroversion | 0.72 | 0.59 | 0.88 | **0.00** | Parent domain |
| Increasing | Maternal personality: Agreeableness | 0.74 | 0.61 | 0.89 | **0.00** | Parent domain |
| Increasing | Maternal personality: Neuroticism | 1.33 | 1.09 | 1.62 | **0.00** | Parent domain |
| Increasing | Parental antisocial behaviour | 1.42 | 1.18 | 1.71 | **0.00** | Parent domain |
| Increasing | Parental alcoholism | 1.15 | 0.98 | 1.34 | 0.08 | Parent domain |
| Decreasing | Child IQ | 0.56 | 0.46 | 0.69 | **0.00** | Neuro domain |
| Decreasing | Child executive function | 0.66 | 0.55 | 0.79 | **0.00** | Neuro domain |
| Decreasing | Child theory of mind | 0.61 | 0.49 | 0.77 | **0.00** | Neuro domain |
| Increasing | Child IQ | 0.64 | 0.51 | 0.79 | **0.00** | Neuro domain |
| Increasing | Child executive function | 0.88 | 0.73 | 1.06 | 0.19 | Neuro domain |
| Increasing | Child theory of mind | 0.65 | 0.52 | 0.81 | **0.00** | Neuro domain |
| Decreasing | Child externalising behaviours | 2.47 | 2.11 | 2.89 | **0.00** | Emo/behave domain |
| Decreasing | Child internalising behaviours | 3.37 | 2.79 | 4.08 | **0.00** | Emo/behave domain |
| Decreasing | Child ADHD behaviours | 2.85 | 2.38 | 3.41 | **0.00** | Emo/behave domain |
| Decreasing | Child prosocial behaviours | 0.42 | 0.34 | 0.51 | **0.00** | Emo/behave domain |
| Increasing | Child externalising behaviours | 2.06 | 1.77 | 2.39 | **0.00** | Emo/behave domain |
| Increasing | Child internalising behaviours | 1.66 | 1.38 | 1.99 | **0.00** | Emo/behave domain |
| Increasing | Child ADHD behaviours | 2.39 | 2.02 | 2.82 | **0.00** | Emo/behave domain |
| Increasing | Child prosocial behaviours | 0.64 | 0.53 | 0.78 | **0.00** | Emo/behave domain |

### Table 16. Multivariate model (class ~ variable1 + variable2 + variable3)

| **Table 17.** Relative Risk Ratios (RRR) and 95% confidence intervals for multivariate antecedent multinomial regression model | | | | | | |
| --- | --- | --- | --- | --- | --- | --- |
| **Class** | **Variable** | **RRR** | **95% CI low** | **95% CI high** | **p value** | **Domain** |
| Decreasing | ACORN | 0.91 | 0.61 | 1.35 | 0.64 | Social domain |
| Decreasing | Number of children eligible for free school meals | 1.06 | 0.73 | 1.54 | 0.77 | Social domain |
| Decreasing | Number of children in school | 1.43 | 1.07 | 1.93 | **0.02** | Social domain |
| Decreasing | Problems with neighbours | 0.84 | 0.59 | 1.19 | 0.32 | Social domain |
| Decreasing | SES: Low | 1.00 | 0.48 | 2.12 | 0.99 | Social domain |
| Decreasing | Vandalism | 0.88 | 0.63 | 1.23 | 0.46 | Social domain |
| Increasing | ACORN | 0.94 | 0.67 | 1.32 | 0.71 | Social domain |
| Increasing | Number of children eligible for free school meals | 1.02 | 0.73 | 1.42 | 0.91 | Social domain |
| Increasing | Number of children in school | 1.07 | 0.81 | 1.43 | 0.62 | Social domain |
| Increasing | Problems with neighbours | 1.17 | 0.89 | 1.55 | 0.25 | Social domain |
| Increasing | SES: Low | 1.48 | 0.77 | 2.83 | 0.24 | Social domain |
| Increasing | Vandalism | 0.80 | 0.58 | 1.10 | 0.17 | Social domain |
| Decreasing | Any domestic violence: Yes | 0.93 | 0.49 | 1.79 | 0.84 | Home domain |
| Decreasing | Child harm: Harmed | 1.14 | 0.56 | 2.33 | 0.71 | Home domain |
| Decreasing | Maternal warmth | 1.06 | 0.81 | 1.37 | 0.68 | Home domain |
| Decreasing | Mum not lived with biological dad since birth: Yes | 0.89 | 0.34 | 2.32 | 0.81 | Home domain |
| Decreasing | Social support | 0.87 | 0.64 | 1.18 | 0.37 | Home domain |
| Decreasing | Total activities with mum | 1.04 | 0.76 | 1.43 | 0.79 | Home domain |
| Decreasing | Total siblings | 1.15 | 0.91 | 1.45 | 0.25 | Home domain |
| Increasing | Any domestic violence: Yes | 0.75 | 0.41 | 1.38 | 0.36 | Home domain |
| Increasing | Child harm: Harmed | 1.19 | 0.57 | 2.48 | 0.65 | Home domain |
| Increasing | Maternal warmth | 1.00 | 0.77 | 1.30 | 1.00 | Home domain |
| Increasing | Mum not lived with biological dad since birth: Yes | 1.10 | 0.42 | 2.87 | 0.85 | Home domain |
| Increasing | Social support | 0.92 | 0.72 | 1.18 | 0.53 | Home domain |
| Increasing | Total activities with mum | 0.79 | 0.60 | 1.03 | 0.08 | Home domain |
| Increasing | Total siblings | 0.82 | 0.61 | 1.11 | 0.21 | Home domain |
| Decreasing | Maternal lifetime depression: Yes | 1.08 | 0.58 | 1.99 | 0.81 | Parent domain |
| Decreasing | Maternal personality: Agreeableness | 0.97 | 0.64 | 1.46 | 0.88 | Parent domain |
| Decreasing | Maternal personality: Conscientiousness | 0.85 | 0.60 | 1.20 | 0.35 | Parent domain |
| Decreasing | Maternal personality: Extroversion | 0.84 | 0.58 | 1.22 | 0.36 | Parent domain |
| Decreasing | Maternal personality: Neuroticism | 1.01 | 0.65 | 1.57 | 0.95 | Parent domain |
| Decreasing | Maternal personality: Openness | 1.67 | 1.12 | 2.50 | **0.01** | Parent domain |
| Decreasing | Parental alcoholism | 1.09 | 0.80 | 1.50 | 0.57 | Parent domain |
| Decreasing | Parental antisocial behaviour | 0.93 | 0.63 | 1.36 | 0.69 | Parent domain |
| Increasing | Maternal lifetime depression: Yes | 1.12 | 0.62 | 2.02 | 0.70 | Parent domain |
| Increasing | Maternal personality: Agreeableness | 0.93 | 0.67 | 1.28 | 0.65 | Parent domain |
| Increasing | Maternal personality: Conscientiousness | 1.00 | 0.69 | 1.43 | 0.98 | Parent domain |
| Increasing | Maternal personality: Extroversion | 1.05 | 0.71 | 1.56 | 0.82 | Parent domain |
| Increasing | Maternal personality: Neuroticism | 0.93 | 0.66 | 1.31 | 0.69 | Parent domain |
| Increasing | Maternal personality: Openness | 0.94 | 0.60 | 1.48 | 0.80 | Parent domain |
| Increasing | Parental alcoholism | 0.81 | 0.63 | 1.04 | 0.10 | Parent domain |
| Increasing | Parental antisocial behaviour | 0.97 | 0.66 | 1.44 | 0.90 | Parent domain |
| Decreasing | Child executive function | 0.79 | 0.62 | 1.00 | 0.05 | Neuro domain |
| Decreasing | Child IQ | 0.96 | 0.71 | 1.28 | 0.77 | Neuro domain |
| Decreasing | Child theory of mind | 1.02 | 0.74 | 1.41 | 0.90 | Neuro domain |
| Increasing | Child executive function | 1.30 | 1.02 | 1.66 | **0.04** | Neuro domain |
| Increasing | Child IQ | 1.00 | 0.75 | 1.34 | 1.00 | Neuro domain |
| Increasing | Child theory of mind | 0.82 | 0.63 | 1.07 | 0.15 | Neuro domain |
| Decreasing | Child ADHD behaviours | 1.78 | 1.25 | 2.54 | **0.00** | Emo/behave domain |
| Decreasing | Child externalising behaviours | 1.15 | 0.79 | 1.67 | 0.46 | Emo/behave domain |
| Decreasing | Child internalising behaviours | 3.02 | 2.29 | 3.97 | **0.00** | Emo/behave domain |
| Decreasing | Child prosocial behaviours | 0.59 | 0.43 | 0.81 | **0.00** | Emo/behave domain |
| Increasing | Child ADHD behaviours | 1.90 | 1.40 | 2.57 | **0.00** | Emo/behave domain |
| Increasing | Child externalising behaviours | 1.30 | 0.94 | 1.78 | 0.11 | Emo/behave domain |
| Increasing | Child internalising behaviours | 1.22 | 0.95 | 1.56 | 0.12 | Emo/behave domain |
| Increasing | Child prosocial behaviours | 1.01 | 0.77 | 1.33 | 0.95 | Emo/behave domain |

## Multiple testing (FDR) correction

The false discovery rate (FDR) multiple testing correction were applied to all models (Benjamini & Hochberg, 1995). Univariate antecedent models were corrected for 28 tests (28 antecedent variables). Univariate logistic outcome models were corrected for 11 tests and univariate linear outcome models were corrected for 20 tests (total of 31 outcome variables). All variables remained significant, apart from the association between urban residence (ACORN) and the increasing trajectory of social isolation (RRR=1.27 95% CI=1, 1.60, *adjusted* *p*=0.0687).

# Appendix S8. Associations with outcomes at age 18

## Figure 5. Outcome variables multicollinearity

Excluding variables that represent clinical cut off variables of continuous measures; no measures breached the multicollinearity threshold (r>0.9).

##
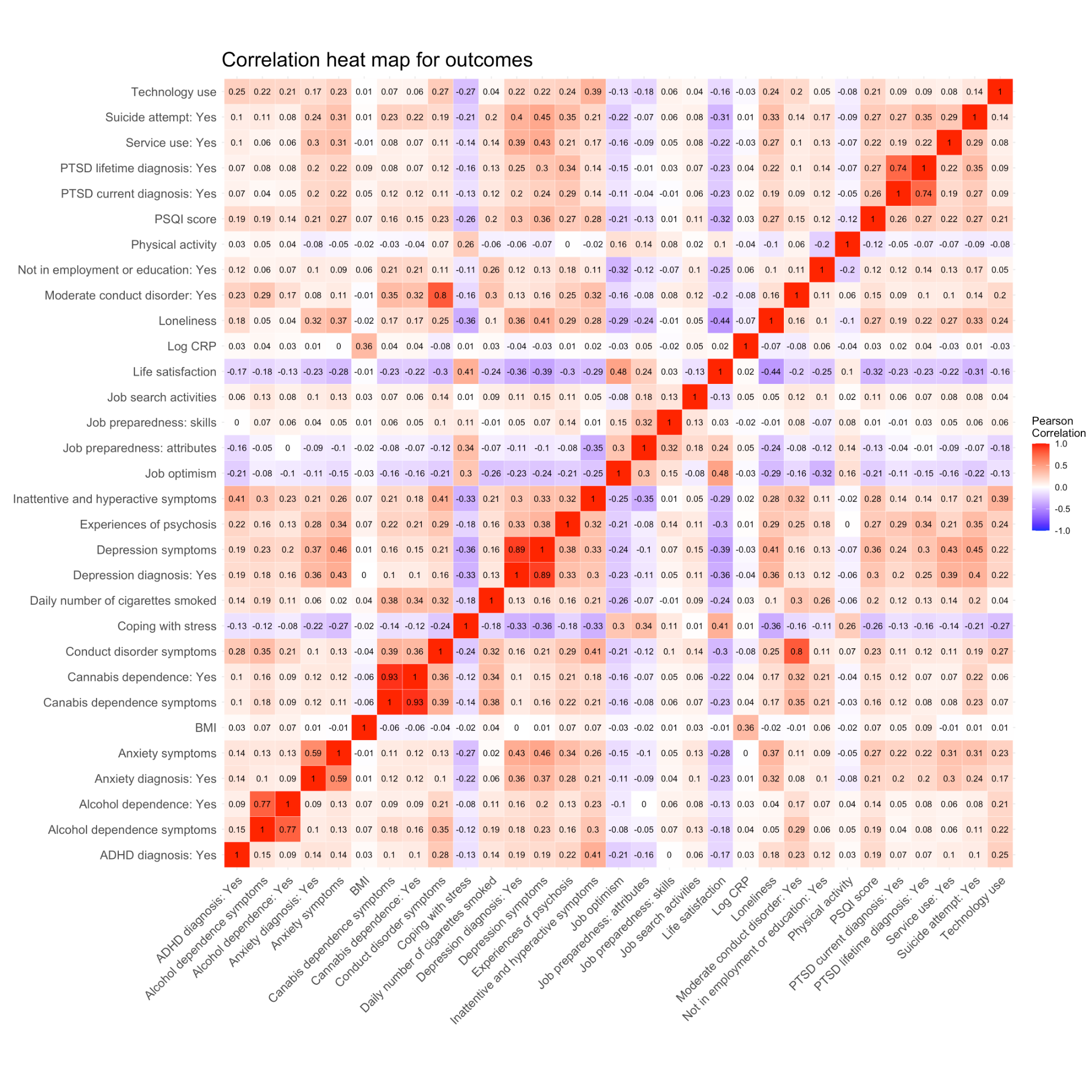


##

##

##

##

##

##

##

##

##

##

##

##

##

##

##

##

##

##

##

##

##

##

##

##

##

##

##

##

## Table 18. Outcome logistic regression models with Odds Ratio (OR) estimates and 95% confidence intervals (binary.variable ~ class)

| **Class** | **Variable** | **OR** | **95% CI low** | **95% CI high** | **p value** | **Domain** |
| --- | --- | --- | --- | --- | --- | --- |
| Increasing | Depression diagnosis | 1.32 | 0.81 | 2.15 | 0.26 | Mental health domain |
| Decreasing | Depression diagnosis | 1.57 | 1.00 | 2.45 | **0.05** | Mental health domain |
| Increasing | Anxiety diagnosis | 1.56 | 0.79 | 3.10 | 0.20 | Mental health domain |
| Decreasing | Anxiety diagnosis | 1.16 | 0.56 | 2.43 | 0.69 | Mental health domain |
| Increasing | ADHD diagnosis | 2.15 | 1.15 | 4.01 | **0.02** | Mental health domain |
| Decreasing | ADHD diagnosis | 1.78 | 0.95 | 3.32 | 0.07 | Mental health domain |
| Increasing | Moderate conduct disorder | 1.91 | 1.16 | 3.15 | **0.01** | Mental health domain |
| Decreasing | Moderate conduct disorder | 1.21 | 0.70 | 2.08 | 0.50 | Mental health domain |
| Increasing | Alcohol dependence | 0.73 | 0.36 | 1.45 | 0.36 | Mental health domain |
| Decreasing | Alcohol dependence | 0.84 | 0.46 | 1.55 | 0.58 | Mental health domain |
| Increasing | Cannabis dependence | 2.22 | 1.08 | 4.54 | **0.03** | Mental health domain |
| Decreasing | Cannabis dependence | 2.11 | 1.01 | 4.39 | **0.05** | Mental health domain |
| Increasing | PTSD lifetime diagnosis | 1.41 | 0.69 | 2.87 | 0.35 | Mental health domain |
| Decreasing | PTSD lifetime diagnosis | 1.12 | 0.54 | 2.34 | 0.75 | Mental health domain |
| Increasing | PTSD current diagnosis | 1.28 | 0.47 | 3.53 | 0.63 | Mental health domain |
| Decreasing | PTSD current diagnosis | 0.84 | 0.30 | 2.38 | 0.75 | Mental health domain |
| Increasing | Suicide attempt | 1.99 | 1.16 | 3.39 | **0.01** | Mental health domain |
| Decreasing | Suicide attempt | 1.69 | 1.02 | 2.79 | **0.04** | Mental health domain |
| Increasing | Service use | 1.91 | 1.10 | 3.32 | **0.02** | Mental health domain |
| Decreasing | Service use | 1.06 | 0.58 | 1.95 | 0.85 | Mental health domain |
| Increasing | Not in employment or education | 2.07 | 1.24 | 3.47 | **0.01** | Employment domain |
| Decreasing | Not in employment or education | 1.63 | 0.91 | 2.95 | 0.10 | Employment domain |

## Table 19. Outcome linear regression models with Beta estimates and 95% confidence intervals (linear.variable ~ class)

| **Class** | **Variable** | **Beta estimate** | **95% CI low** | **95% CI high** | **p value** | **Domain** |
| --- | --- | --- | --- | --- | --- | --- |
| Increasing | Depression symptoms | 0.20 | -0.02 | 0.43 | 0.08 | Mental health domain |
| Decreasing | Depression symptoms | 0.26 | 0.05 | 0.47 | **0.02** | Mental health domain |
| Increasing | Anxiety symptoms | 0.10 | -0.13 | 0.32 | 0.39 | Mental health domain |
| Decreasing | Anxiety symptoms | 0.07 | -0.14 | 0.27 | 0.52 | Mental health domain |
| Increasing | Inattentive and hyperactive symptoms | 0.57 | 0.36 | 0.79 | **0.00** | Mental health domain |
| Decreasing | Inattentive and hyperactive symptoms | -0.03 | -0.23 | 0.18 | 0.81 | Mental health domain |
| Increasing | Conduct disorder symptoms | 0.41 | 0.15 | 0.66 | **0.00** | Mental health domain |
| Decreasing | Conduct disorder symptoms | 0.06 | -0.16 | 0.28 | 0.60 | Mental health domain |
| Increasing | Alcohol dependence symptoms | -0.11 | -0.29 | 0.08 | 0.24 | Mental health domain |
| Decreasing | Alcohol dependence symptoms | 0.01 | -0.23 | 0.25 | 0.92 | Mental health domain |
| Increasing | Cannabis dependence symptoms | 0.23 | -0.03 | 0.49 | 0.09 | Mental health domain |
| Decreasing | Cannabis dependence symptoms | 0.30 | -0.01 | 0.62 | 0.06 | Mental health domain |
| Increasing | Experiences of psychosis | 0.35 | 0.09 | 0.60 | **0.01** | Mental health domain |
| Decreasing | Experiences of psychosis | 0.31 | 0.07 | 0.55 | **0.01** | Mental health domain |
| Increasing | BMI | 0.22 | -0.04 | 0.48 | 0.09 | Physical health domain |
| Decreasing | BMI | 0.03 | -0.15 | 0.21 | 0.74 | Physical health domain |
| Increasing | Log CRP | 0.07 | -0.16 | 0.30 | 0.55 | Physical health domain |
| Decreasing | Log CRP | 0.17 | -0.07 | 0.41 | 0.16 | Physical health domain |
| Increasing | Physical activity | -0.28 | -0.48 | -0.07 | **0.01** | Physical health domain |
| Decreasing | Physical activity | -0.43 | -0.60 | -0.25 | **0.00** | Physical health domain |
| Increasing | Daily number of cigarettes smoked | 0.46 | 0.08 | 0.84 | **0.02** | Physical health domain |
| Decreasing | Daily number of cigarettes smoked | 0.19 | -0.01 | 0.39 | **0.06** | Physical health domain |
| Increasing | Loneliness | 0.37 | 0.13 | 0.61 | **0.00** | Coping and functioning domain |
| Decreasing | Loneliness | 0.19 | -0.03 | 0.42 | 0.08 | Coping and functioning domain |
| Increasing | Life satisfaction | -0.29 | -0.52 | -0.06 | **0.01** | Coping and functioning domain |
| Decreasing | Life satisfaction | -0.20 | -0.42 | 0.02 | 0.08 | Coping and functioning domain |
| Increasing | Technology use | 0.24 | 0.02 | 0.46 | **0.03** | Coping and functioning domain |
| Decreasing | Technology use | 0.18 | -0.03 | 0.39 | 0.10 | Coping and functioning domain |
| Increasing | Coping with stress | -0.34 | -0.55 | -0.12 | **0.00** | Coping and functioning domain |
| Decreasing | Coping with stress | -0.08 | -0.27 | 0.12 | 0.45 | Coping and functioning domain |
| Increasing | PSQI score | 0.21 | 0.01 | 0.41 | **0.04** | Coping and functioning domain |
| Decreasing | PSQI score | 0.06 | -0.14 | 0.25 | 0.57 | Coping and functioning domain |
| Increasing | Job preparedness: skills | 0.01 | -0.23 | 0.24 | 0.96 | Employment domain |
| Decreasing | Job preparedness: skills | -0.03 | -0.24 | 0.18 | 0.76 | Employment domain |
| Increasing | Job preparedness: attributes | -0.37 | -0.62 | -0.13 | **0.00** | Employment domain |
| Decreasing | Job preparedness: attributes | -0.12 | -0.32 | 0.09 | 0.27 | Employment domain |
| Increasing | Job optimism | -0.58 | -0.80 | -0.36 | **0.00** | Employment domain |
| Decreasing | Job optimism | -0.27 | -0.47 | -0.07 | **0.01** | Employment domain |
| Increasing | Job search activities | -0.06 | -0.27 | 0.15 | 0.57 | Employment domain |
| Decreasing | Job search activities | 0.06 | -0.16 | 0.29 | 0.58 | Employment domain |

##

## Multiple testing (FDR) correction

The false discovery rate (FDR) multiple testing correction were applied to all models (Benjamini & Hochberg, 1995). Univariate logistic outcome models were corrected for 11 tests and univariate linear outcome models were corrected for 20 tests (total of 31 outcome variables).

### Logistic regression models

For outcomes associated with the decreasing trajectory of social isolation, cannabis dependence (OR=2.11, 95% CI=1.01, 4.39, *adjusted* *p*=0.187) and suicide attempt (OR=1.69, 95% CI=1.01, 2.79, *adjusted* *p*=0.187) became non-significant.

For outcomes associated with the increasing trajectory of social isolation, cannabis dependence (OR=2.22, 95% CI=1.08, 4.54, *adjusted* *p*=0.054) became non-significant.

### Linear regression models

For outcomes associated with the decreasing trajectory of social isolation, depression symptoms (β=0.26, *adjusted* *p*=0.086), experiences of psychosis (β=0.31, *adjusted* *p*=0.072), and job optimism (β=-0.27, *adjusted* *p*=0.072) became non-significant.

For outcomes associated with the increasing trajectory of social isolation, technology use (β=0.24, *adjusted* *p*=0.055) and PSQI score (β=0.21, *adjusted* *p*=0.061) became non-significant.

# Appendix S9. Association with outcomes at age 18 whilst controlling for antecedents at age 5

## Table 20. Logistic outcome models, controlling for antecedents (logistic.variable ~ class + antecedent1 + antecedent2 + antecedent3)

| **Class** | **Variable** | **OR** | **95% CI low** | **95% CI high** | **p value** | **Domain** |
| --- | --- | --- | --- | --- | --- | --- |
| Increasing | Depression diagnosis | 1.15 | 0.67 | 1.96 | 0.61 | Mental health domain |
| Decreasing | Depression diagnosis | 1.28 | 0.75 | 2.18 | 0.37 | Mental health domain |
| Increasing | Anxiety diagnosis | 1.18 | 0.52 | 2.67 | 0.69 | Mental health domain |
| Decreasing | Anxiety diagnosis | 1.09 | 0.50 | 2.38 | 0.83 | Mental health domain |
| Increasing | ADHD diagnosis | 1.19 | 0.60 | 2.36 | 0.61 | Mental health domain |
| Decreasing | ADHD diagnosis | 0.78 | 0.36 | 1.68 | 0.52 | Mental health domain |
| Increasing | Moderate conduct disorder | 1.56 | 0.90 | 2.69 | 0.11 | Mental health domain |
| Decreasing | Moderate conduct disorder | 0.90 | 0.47 | 1.73 | 0.75 | Mental health domain |
| Increasing | Alcohol dependence | 0.74 | 0.35 | 1.57 | 0.43 | Mental health domain |
| Decreasing | Alcohol dependence | 0.86 | 0.43 | 1.74 | 0.68 | Mental health domain |
| Increasing | Cannabis dependence | 1.76 | 0.77 | 4.04 | 0.18 | Mental health domain |
| Decreasing | Cannabis dependence | 1.34 | 0.59 | 3.03 | 0.48 | Mental health domain |
| Increasing | PTSD lifetime diagnosis | 1.32 | 0.60 | 2.92 | 0.49 | Mental health domain |
| Decreasing | PTSD lifetime diagnosis | 1.03 | 0.44 | 2.42 | 0.94 | Mental health domain |
| Increasing | PTSD current diagnosis | 1.14 | 0.35 | 3.73 | 0.83 | Mental health domain |
| Decreasing | PTSD current diagnosis | 0.75 | 0.22 | 2.56 | 0.65 | Mental health domain |
| Increasing | Suicide attempt | 1.77 | 1.00 | 3.14 | **0.05** | Mental health domain |
| Decreasing | Suicide attempt | 1.47 | 0.82 | 2.62 | 0.19 | Mental health domain |
| Increasing | Service use | 1.62 | 0.90 | 2.91 | 0.11 | Mental health domain |
| Decreasing | Service use | 0.74 | 0.37 | 1.51 | 0.41 | Mental health domain |
| Increasing | Not in employment or education | 1.35 | 0.77 | 2.38 | 0.30 | Employment domain |
| Decreasing | Not in employment or education | 0.75 | 0.38 | 1.48 | 0.41 | Employment domain |

## Table 21. Linear outcome models, controlling for antecedents (linear.variable ~ class + antecedent1 + antecedent2 + antecedent3)

| **Class** | **Variable** | **Beta estimate** | **95% CI low** | **95% CI high** | **p value** | **Domain** |
| --- | --- | --- | --- | --- | --- | --- |
| Increasing | Depression symptoms | 0.14 | -0.10 | 0.38 | 0.26 | Mental health domain |
| Decreasing | Depression symptoms | 0.15 | -0.09 | 0.40 | 0.21 | Mental health domain |
| Increasing | Anxiety symptoms | -0.01 | -0.26 | 0.23 | 0.91 | Mental health domain |
| Decreasing | Anxiety symptoms | -0.07 | -0.31 | 0.17 | 0.55 | Mental health domain |
| Increasing | Inattentive and hyperactive symptoms | 0.35 | 0.12 | 0.58 | **0.00** | Mental health domain |
| Decreasing | Inattentive and hyperactive symptoms | -0.24 | -0.47 | -0.02 | **0.03** | Mental health domain |
| Increasing | Conduct disorder symptoms | 0.30 | 0.03 | 0.57 | **0.03** | Mental health domain |
| Decreasing | Conduct disorder symptoms | -0.10 | -0.33 | 0.14 | 0.42 | Mental health domain |
| Increasing | Alcohol dependence symptoms | -0.15 | -0.34 | 0.04 | 0.12 | Mental health domain |
| Decreasing | Alcohol dependence symptoms | -0.04 | -0.31 | 0.24 | 0.79 | Mental health domain |
| Increasing | Cannabis dependence symptoms | 0.14 | -0.15 | 0.43 | 0.34 | Mental health domain |
| Decreasing | Cannabis dependence symptoms | 0.17 | -0.15 | 0.49 | 0.30 | Mental health domain |
| Increasing | Experiences of psychosis | 0.20 | -0.07 | 0.47 | 0.14 | Mental health domain |
| Decreasing | Experiences of psychosis | 0.16 | -0.11 | 0.42 | 0.26 | Mental health domain |
| Increasing | BMI | 0.20 | -0.08 | 0.48 | 0.16 | Physical health domain |
| Decreasing | BMI | 0.05 | -0.17 | 0.26 | 0.67 | Physical health domain |
| Increasing | Log CRP | 0.08 | -0.17 | 0.33 | 0.52 | Physical health domain |
| Decreasing | Log CRP | 0.18 | -0.09 | 0.45 | 0.19 | Physical health domain |
| Increasing | Physical activity | -0.21 | -0.43 | 0.01 | **0.06** | Physical health domain |
| Decreasing | Physical activity | -0.27 | -0.48 | -0.06 | **0.01** | Physical health domain |
| Increasing | Daily number of cigarettes smoked | 0.37 | -0.03 | 0.77 | 0.07 | Physical health domain |
| Decreasing | Daily number of cigarettes smoked | -0.03 | -0.26 | 0.19 | 0.77 | Physical health domain |
| Increasing | Loneliness | 0.29 | 0.05 | 0.53 | **0.02** | Coping and functioning domain |
| Decreasing | Loneliness | 0.09 | -0.16 | 0.35 | 0.47 | Coping and functioning domain |
| Increasing | Life satisfaction | -0.17 | -0.41 | 0.07 | 0.17 | Coping and functioning domain |
| Decreasing | Life satisfaction | -0.04 | -0.29 | 0.22 | 0.77 | Coping and functioning domain |
| Increasing | Technology use | 0.13 | -0.10 | 0.35 | 0.26 | Coping and functioning domain |
| Decreasing | Technology use | 0.07 | -0.17 | 0.31 | 0.58 | Coping and functioning domain |
| Increasing | Coping with stress | -0.21 | -0.45 | 0.02 | 0.07 | Coping and functioning domain |
| Decreasing | Coping with stress | 0.10 | -0.12 | 0.33 | 0.36 | Coping and functioning domain |
| Increasing | PSQI score | 0.13 | -0.08 | 0.35 | 0.22 | Coping and functioning domain |
| Decreasing | PSQI score | -0.05 | -0.28 | 0.18 | 0.66 | Coping and functioning domain |
| Increasing | Job preparedness: skills | 0.13 | -0.12 | 0.37 | 0.32 | Employment domain |
| Decreasing | Job preparedness: skills | 0.15 | -0.09 | 0.39 | 0.22 | Employment domain |
| Increasing | Job preparedness: attributes | -0.19 | -0.46 | 0.07 | 0.15 | Employment domain |
| Decreasing | Job preparedness: attributes | 0.16 | -0.08 | 0.40 | 0.19 | Employment domain |
| Increasing | Job optimism | -0.40 | -0.64 | -0.16 | **0.00** | Employment domain |
| Decreasing | Job optimism | -0.03 | -0.26 | 0.19 | 0.78 | Employment domain |
| Increasing | Job search activities | -0.11 | -0.34 | 0.12 | 0.35 | Employment domain |
| Decreasing | Job search activities | 0.09 | -0.16 | 0.35 | 0.46 | Employment domain |

# Appendix S10. Sex interactions

When sex and a sex*class interaction was added to the outcome logistic and linear regression models, significant interaction terms were found for outcomes of service use (increasing, p=0.046), not in employment or education (increasing, p=0.018; decreasing, p=0.004), cannabis dependence symptoms (increasing, p=0.048), CRP (increasing, p=0.016), BMI (increasing, p<0.001), and PSQI score (decreasing, p=0.025).

## Not in employment or education

| **Table 22.** Logistic regression for NEET and social isolation class, stratified by sex | | | | | |
| --- | --- | --- | --- | --- | --- |
| **Sex** | **Class** | **OR** | **OR 25% CI** | **OR 95% CI** | ***p* value** |
| Male | Increasing | 3.488 | 1.870 | 6.504 | <0.001 |
| Male | Decreasing | 0.557 | 0.203 | 1.528 | 0.256 |
| Female | Increasing | 0.773 | 0.304 | 1.964 | 0.589 |
| Female | Decreasing | 3.466 | 1.574 | 7.635 | 0.002 |

## Service use

| **Table 23.** Logistic regression for service use and social isolation class, stratified by sex | | | | | |
| --- | --- | --- | --- | --- | --- |
| Sex | Class | OR | OR 25% CI | OR 95% CI | *p* value |
| Male | Increasing | 3.134 | 1.571 | 6.253 | 0.001 |
| Male | Decreasing | 1.269 | 0.532 | 3.029 | 0.590 |
| Female | Increasing | 1.032 | 0.440 | 2.420 | 0.947 |
| Female | Decreasing | 0.929 | 0.406 | 2.126 | 0.862 |

## Cannabis dependence

| **Table 24.** Linear regression for cannabis dependence and social isolation class, stratified by sex | | | | | |
| --- | --- | --- | --- | --- | --- |
| Sex | Class | Estimate | 25% CI | 95% CI | *p* value |
| Male | Increasing | 0.4557305 | 0.0399015 | 0.8715596 | 0.0317446 |
| Male | Decreasing | 0.3522822 | -0.1327783 | 0.8373427 | 0.1544138 |
| Female | Increasing | 0.000 | -0.1783056 | 0.1783056 | 1.0000000 |
| Female | Decreasing | 0.275 | -0.0734461 | 0.6234461 | 0.1217771 |

## BMI

| **Table 25.** Linear regression for body mass index (BMI) and social isolation class, stratified by sex | | | | | |
| --- | --- | --- | --- | --- | --- |
| Sex | Class | Estimate | 25% CI | 95% CI | *p* value |
| Male | Increasing | -0.694 | -1.823 | 0.433 | 0.227 |
| Male | Decreasing | -0.217 | -1.373 | 0.938 | 0.711 |
| Female | Increasing | 4.011 | 1.5969 | 6.425 | 0.001 |
| Female | Decreasing | 0.746 | -0.557 | 2.050 | 0.261 |

## CRP

| **Table 26.** Linear regression for C-reactive protein (CRP) and social isolation class, stratified by sex | | | | | |
| --- | --- | --- | --- | --- | --- |
| Sex | Class | Estimate | 25% CI | 95% CI | *p* value |
| Male | Increasing | -0.233 | -0.644 | 0.178 | 0.265 |
| Male | Decreasing | -0.013 | -0.363 | 0.335 | 0.938 |
| Female | Increasing | 0.531 | 0.060 | 1.002 | 0.027 |
| Female | Decreasing | 0.602 | -0.015 | 1.220 | 0.055 |

## PSQI

| **Table 27.** Linear regression for sleep quality (PSQI) and social isolation class, stratified by sex | | | | | |
| --- | --- | --- | --- | --- | --- |
| Sex | Class | Estimate | 25% CI | 95% CI | *p* value |
| Male | Increasing | 0.771 | -0.024 | 1.567 | 0.057 |
| Male | Decreasing | 0.874 | 0.131 | 1.617 | 0.021 |
| Female | Increasing | 0.899 | -0.103 | 1.901 | 0.078 |
| Female | Decreasing | -0.520 | -1.493 | 0.452 | 0.296 |

# Appendix S11. Posterior probability sensitivity analysis

As a sensitivity check for correct classification of the social isolation classes. All participants who had a posterior probability of under 0.8 for their membership to one of the classes were dropped from the sample. Frequency statistics are given in **Table 28**. The low stable class dropped 42 people, with an average posterior probability of 0.66 and a minimum probability of 0.48. The increasing class dropped 24 people, with an average probability of 0.64 and minimum probability of 0.49. The decreasing class dropped 25 people, with an average probability of 0.63 and minimum probability of 0.51.

| **Table 28.** Frequencies for each class once participants with <0.8 posterior probability have been removed from the sample. | | | |
| --- | --- | --- | --- |
|  | **Frequency** | **%** | **% Cumulative** |
| **Low stable** | 1967 | 91.87 | 91.87 |
| **Increasing** | 82 | 3.83 | 95.7 |
| **Decreasing** | 92 | 4.30 | 100 |
| **Total** | 2141 | 100 | 100 |

## Antecedents

### Univariate models

| **Table 29.** Univariate multinomial regression models for antecedents: posterior probability sensitivity analysis (N=2141) | | | | | | |
| --- | --- | --- | --- | --- | --- | --- |
| **Class** | **Variable** | **RRR** | **95% CI low** | **95% CI high** | **p value** | **Domain** |
| Decreasing | SES: Low | 1.89 | 1.21 | 2.94 | 0.00 | Social domain |
| Decreasing | ACORN | 1.24 | 1.00 | 1.54 | 0.05 | Social domain |
| Decreasing | Vandalism | 1.11 | 0.92 | 1.36 | 0.28 | Social domain |
| Decreasing | Problems with neighbours | 1.22 | 1.02 | 1.46 | 0.03 | Social domain |
| Decreasing | Number of children in school | 1.19 | 0.92 | 1.53 | 0.18 | Social domain |
| Decreasing | Number of children eligible for free school meals | 1.34 | 1.11 | 1.62 | 0.00 | Social domain |
| Increasing | SES: Low | 2.72 | 1.70 | 4.38 | 0.00 | Social domain |
| Increasing | ACORN | 1.16 | 0.90 | 1.50 | 0.24 | Social domain |
| Increasing | Vandalism | 1.06 | 0.85 | 1.31 | 0.61 | Social domain |
| Increasing | Problems with neighbours | 1.28 | 1.09 | 1.52 | 0.00 | Social domain |
| Increasing | Number of children in school | 1.07 | 0.82 | 1.41 | 0.60 | Social domain |
| Increasing | Number of children eligible for free school meals | 1.13 | 0.93 | 1.37 | 0.22 | Social domain |
| Decreasing | Child harm: Harmed | 3.00 | 1.86 | 4.84 | 0.00 | Home domain |
| Decreasing | Total siblings | 1.37 | 1.14 | 1.63 | 0.00 | Home domain |
| Decreasing | Social support | 0.69 | 0.55 | 0.87 | 0.00 | Home domain |
| Decreasing | Total activities with mum | 0.66 | 0.53 | 0.83 | 0.00 | Home domain |
| Decreasing | Mum not lived with biological dad since birth: Yes | 2.25 | 1.07 | 4.73 | 0.03 | Home domain |
| Decreasing | Any domestic violence: Yes | 1.72 | 1.10 | 2.68 | 0.02 | Home domain |
| Decreasing | Maternal warmth | 0.64 | 0.51 | 0.80 | 0.00 | Home domain |
| Increasing | Child harm: Harmed | 1.94 | 1.10 | 3.45 | 0.02 | Home domain |
| Increasing | Total siblings | 1.04 | 0.80 | 1.35 | 0.76 | Home domain |
| Increasing | Social support | 0.70 | 0.55 | 0.88 | 0.00 | Home domain |
| Increasing | Total activities with mum | 0.65 | 0.52 | 0.82 | 0.00 | Home domain |
| Increasing | Mum not lived with biological dad since birth: Yes | 3.13 | 1.58 | 6.21 | 0.00 | Home domain |
| Increasing | Any domestic violence: Yes | 1.25 | 0.77 | 2.01 | 0.37 | Home domain |
| Increasing | Maternal warmth | 0.57 | 0.45 | 0.71 | 0.00 | Home domain |
| Decreasing | Maternal lifetime depression: Yes | 1.86 | 1.20 | 2.88 | 0.01 | Parent domain |
| Decreasing | Maternal personality: Openness | 0.81 | 0.65 | 1.01 | 0.07 | Parent domain |
| Decreasing | Maternal personality: Conscientiousness | 0.60 | 0.48 | 0.74 | 0.00 | Parent domain |
| Decreasing | Maternal personality: Extroversion | 0.77 | 0.63 | 0.95 | 0.02 | Parent domain |
| Decreasing | Maternal personality: Agreeableness | 0.68 | 0.53 | 0.86 | 0.00 | Parent domain |
| Decreasing | Maternal personality: Neuroticism | 1.42 | 1.14 | 1.77 | 0.00 | Parent domain |
| Decreasing | Parental antisocial behaviour | 1.65 | 1.40 | 1.95 | 0.00 | Parent domain |
| Decreasing | Parental alcoholism | 1.37 | 1.16 | 1.62 | 0.00 | Parent domain |
| Increasing | Maternal lifetime depression: Yes | 1.39 | 0.86 | 2.23 | 0.18 | Parent domain |
| Increasing | Maternal personality: Openness | 0.65 | 0.52 | 0.83 | 0.00 | Parent domain |
| Increasing | Maternal personality: Conscientiousness | 0.62 | 0.51 | 0.77 | 0.00 | Parent domain |
| Increasing | Maternal personality: Extroversion | 0.76 | 0.61 | 0.94 | 0.01 | Parent domain |
| Increasing | Maternal personality: Agreeableness | 0.70 | 0.58 | 0.85 | 0.00 | Parent domain |
| Increasing | Maternal personality: Neuroticism | 1.41 | 1.13 | 1.76 | 0.00 | Parent domain |
| Increasing | Parental antisocial behaviour | 1.53 | 1.25 | 1.87 | 0.00 | Parent domain |
| Increasing | Parental alcoholism | 1.20 | 1.01 | 1.41 | 0.03 | Parent domain |
| Decreasing | Child IQ | 0.59 | 0.47 | 0.75 | 0.00 | Neuro domain |
| Decreasing | Child executive function | 0.68 | 0.55 | 0.84 | 0.00 | Neuro domain |
| Decreasing | Child theory of mind | 0.61 | 0.48 | 0.78 | 0.00 | Neuro domain |
| Increasing | Child IQ | 0.68 | 0.54 | 0.85 | 0.00 | Neuro domain |
| Increasing | Child executive function | 0.75 | 0.61 | 0.92 | 0.01 | Neuro domain |
| Increasing | Child theory of mind | 0.65 | 0.50 | 0.84 | 0.00 | Neuro domain |
| Decreasing | Child externalising behaviours | 2.62 | 2.21 | 3.11 | 0.00 | Emo/behave domain |
| Decreasing | Child internalising behaviours | 3.59 | 2.95 | 4.38 | 0.00 | Emo/behave domain |
| Decreasing | Child ADHD behaviours | 2.98 | 2.45 | 3.62 | 0.00 | Emo/behave domain |
| Decreasing | Child prosocial behaviours | 0.40 | 0.32 | 0.49 | 0.00 | Emo/behave domain |
| Increasing | Child externalising behaviours | 2.17 | 1.85 | 2.56 | 0.00 | Emo/behave domain |
| Increasing | Child internalising behaviours | 1.76 | 1.44 | 2.15 | 0.00 | Emo/behave domain |
| Increasing | Child ADHD behaviours | 2.55 | 2.13 | 3.05 | 0.00 | Emo/behave domain |
| Increasing | Child prosocial behaviours | 0.61 | 0.49 | 0.75 | 0.00 | Emo/behave domain |

### Multivariate model

| **Table 30.** Multivariate multinomial regression models for antecedents: posterior probability sensitivity analysis (N=2141) | | | | | | |
| --- | --- | --- | --- | --- | --- | --- |
| **Class** | **Variable** | **RRR** | **95% CI low** | **95% CI high** | **p value** | **Domain** |
| Decreasing | ACORN | 0.90 | 0.58 | 1.40 | 0.63 | Social domain |
| Decreasing | Number of children eligible for free school meals | 1.05 | 0.67 | 1.64 | 0.84 | Social domain |
| Decreasing | Number of children in school | 1.69 | 1.18 | 2.40 | 0.00 | Social domain |
| Decreasing | Problems with neighbours | 0.89 | 0.59 | 1.34 | 0.56 | Social domain |
| Decreasing | SES: Low | 0.61 | 0.27 | 1.41 | 0.25 | Social domain |
| Decreasing | Vandalism | 0.80 | 0.54 | 1.18 | 0.26 | Social domain |
| Increasing | ACORN | 0.85 | 0.58 | 1.25 | 0.42 | Social domain |
| Increasing | Number of children eligible for free school meals | 0.97 | 0.71 | 1.34 | 0.87 | Social domain |
| Increasing | Number of children in school | 1.15 | 0.85 | 1.55 | 0.37 | Social domain |
| Increasing | Problems with neighbours | 1.16 | 0.88 | 1.54 | 0.29 | Social domain |
| Increasing | SES: Low | 1.55 | 0.76 | 3.15 | 0.23 | Social domain |
| Increasing | Vandalism | 0.71 | 0.49 | 1.02 | 0.06 | Social domain |
| Decreasing | Any domestic violence: Yes | 0.96 | 0.46 | 2.03 | 0.92 | Home domain |
| Decreasing | Child harm: Harmed | 1.63 | 0.74 | 3.59 | 0.23 | Home domain |
| Decreasing | Maternal warmth | 1.01 | 0.71 | 1.42 | 0.97 | Home domain |
| Decreasing | Mum not lived with biological dad since birth: Yes | 1.81 | 0.73 | 4.47 | 0.20 | Home domain |
| Decreasing | Social support | 0.77 | 0.54 | 1.09 | 0.14 | Home domain |
| Decreasing | Total activities with mum | 0.99 | 0.70 | 1.41 | 0.97 | Home domain |
| Decreasing | Total siblings | 1.42 | 1.11 | 1.82 | 0.01 | Home domain |
| Increasing | Any domestic violence: Yes | 0.55 | 0.28 | 1.08 | 0.08 | Home domain |
| Increasing | Child harm: Harmed | 1.41 | 0.65 | 3.03 | 0.39 | Home domain |
| Increasing | Maternal warmth | 0.84 | 0.65 | 1.09 | 0.19 | Home domain |
| Increasing | Mum not lived with biological dad since birth: Yes | 1.26 | 0.46 | 3.45 | 0.65 | Home domain |
| Increasing | Social support | 0.90 | 0.67 | 1.19 | 0.45 | Home domain |
| Increasing | Total activities with mum | 0.81 | 0.60 | 1.10 | 0.17 | Home domain |
| Increasing | Total siblings | 0.84 | 0.61 | 1.16 | 0.29 | Home domain |
| Decreasing | Maternal lifetime depression: Yes | 1.04 | 0.52 | 2.08 | 0.91 | Parent domain |
| Decreasing | Maternal personality: Agreeableness | 1.11 | 0.69 | 1.78 | 0.67 | Parent domain |
| Decreasing | Maternal personality: Conscientiousness | 0.87 | 0.58 | 1.30 | 0.50 | Parent domain |
| Decreasing | Maternal personality: Extroversion | 0.62 | 0.40 | 0.97 | 0.04 | Parent domain |
| Decreasing | Maternal personality: Neuroticism | 0.91 | 0.54 | 1.53 | 0.73 | Parent domain |
| Decreasing | Maternal personality: Openness | 2.24 | 1.42 | 3.54 | 0.00 | Parent domain |
| Decreasing | Parental alcoholism | 1.17 | 0.81 | 1.70 | 0.39 | Parent domain |
| Decreasing | Parental antisocial behaviour | 0.90 | 0.57 | 1.44 | 0.66 | Parent domain |
| Increasing | Maternal lifetime depression: Yes | 1.55 | 0.82 | 2.93 | 0.18 | Parent domain |
| Increasing | Maternal personality: Agreeableness | 0.92 | 0.65 | 1.31 | 0.64 | Parent domain |
| Increasing | Maternal personality: Conscientiousness | 0.91 | 0.61 | 1.37 | 0.66 | Parent domain |
| Increasing | Maternal personality: Extroversion | 1.07 | 0.69 | 1.67 | 0.76 | Parent domain |
| Increasing | Maternal personality: Neuroticism | 0.91 | 0.64 | 1.29 | 0.58 | Parent domain |
| Increasing | Maternal personality: Openness | 0.96 | 0.59 | 1.58 | 0.88 | Parent domain |
| Increasing | Parental alcoholism | 0.78 | 0.58 | 1.05 | 0.10 | Parent domain |
| Increasing | Parental antisocial behaviour | 1.12 | 0.75 | 1.69 | 0.57 | Parent domain |
| Decreasing | Child executive function | 0.74 | 0.56 | 0.96 | 0.02 | Neuro domain |
| Decreasing | Child IQ | 1.05 | 0.73 | 1.52 | 0.78 | Neuro domain |
| Decreasing | Child theory of mind | 0.92 | 0.64 | 1.33 | 0.67 | Neuro domain |
| Increasing | Child executive function | 1.02 | 0.79 | 1.32 | 0.87 | Neuro domain |
| Increasing | Child IQ | 1.08 | 0.80 | 1.46 | 0.60 | Neuro domain |
| Increasing | Child theory of mind | 0.78 | 0.58 | 1.06 | 0.12 | Neuro domain |
| Decreasing | Child ADHD behaviours | 1.85 | 1.19 | 2.85 | 0.01 | Emo/behave domain |
| Decreasing | Child externalising behaviours | 1.26 | 0.83 | 1.91 | 0.29 | Emo/behave domain |
| Decreasing | Child internalising behaviours | 3.85 | 2.77 | 5.36 | 0.00 | Emo/behave domain |
| Decreasing | Child prosocial behaviours | 0.59 | 0.41 | 0.85 | 0.01 | Emo/behave domain |
| Increasing | Child ADHD behaviours | 2.08 | 1.51 | 2.86 | 0.00 | Emo/behave domain |
| Increasing | Child externalising behaviours | 1.17 | 0.86 | 1.59 | 0.31 | Emo/behave domain |
| Increasing | Child internalising behaviours | 1.31 | 1.01 | 1.71 | 0.04 | Emo/behave domain |
| Increasing | Child prosocial behaviours | 1.02 | 0.74 | 1.39 | 0.92 | Emo/behave domain |
| Note. Significant variables that were not significant in original analysis are marked in red. | | | | | | |

## Outcomes

### Logistic

| **Table 31.** Logistic outcome regressions: posterior probability sensitivity analysis (N=2141) | | | | | | |
| --- | --- | --- | --- | --- | --- | --- |
| **Class** | **Variable** | **OR** | **95% CI low** | **95% CI high** | **p value** | **Domain** |
| Increasing | Depression diagnosis | 1.25 | 0.72 | 2.16 | 0.43 | Mental health domain |
| Decreasing | Depression diagnosis | 1.73 | 1.05 | 2.86 | 0.03 | Mental health domain |
| Increasing | Anxiety diagnosis | 1.40 | 0.63 | 3.10 | 0.41 | Mental health domain |
| Decreasing | Anxiety diagnosis | 1.59 | 0.75 | 3.37 | 0.22 | Mental health domain |
| Increasing | ADHD diagnosis | 2.08 | 1.05 | 4.09 | 0.03 | Mental health domain |
| Decreasing | ADHD diagnosis | 1.82 | 0.90 | 3.70 | 0.10 | Mental health domain |
| Increasing | Moderate conduct disorder | 1.64 | 0.91 | 2.95 | 0.10 | Mental health domain |
| Decreasing | Moderate conduct disorder | 1.48 | 0.81 | 2.69 | 0.20 | Mental health domain |
| Increasing | Alcohol dependence | 0.44 | 0.17 | 1.12 | 0.09 | Mental health domain |
| Decreasing | Alcohol dependence | 0.90 | 0.46 | 1.76 | 0.76 | Mental health domain |
| Increasing | Cannabis dependence | 1.39 | 0.57 | 3.39 | 0.46 | Mental health domain |
| Decreasing | Cannabis dependence | 2.52 | 1.13 | 5.60 | 0.02 | Mental health domain |
| Increasing | PTSD lifetime diagnosis | 1.86 | 0.90 | 3.85 | 0.09 | Mental health domain |
| Decreasing | PTSD lifetime diagnosis | 1.55 | 0.74 | 3.28 | 0.25 | Mental health domain |
| Increasing | PTSD current diagnosis | 1.64 | 0.59 | 4.55 | 0.34 | Mental health domain |
| Decreasing | PTSD current diagnosis | 1.13 | 0.40 | 3.23 | 0.82 | Mental health domain |
| Increasing | Suicide attempt | 2.02 | 1.12 | 3.63 | 0.02 | Mental health domain |
| Decreasing | Suicide attempt | 2.01 | 1.15 | 3.49 | 0.01 | Mental health domain |
| Increasing | Service use | 1.86 | 0.96 | 3.58 | 0.06 | Mental health domain |
| Decreasing | Service use | 1.23 | 0.63 | 2.38 | 0.55 | Mental health domain |
| Increasing | Not in employment or education | 1.81 | 1.03 | 3.17 | 0.04 | Employment domain |
| Decreasing | Not in employment or education | 1.90 | 1.02 | 3.53 | 0.04 | Employment domain |

### Linear

| **Table 32.** Linear outcome regressions: posterior probability sensitivity analysis (N=2141) | | | | | | |
| --- | --- | --- | --- | --- | --- | --- |
| **Class** | **Variable** | **Beta estimate** | **95% CI low** | **95% CI high** | **p value** | **Domain** |
| Increasing | Depression symptoms | 0.19 | -0.06 | 0.45 | 0.14 | Mental health domain |
| Decreasing | Depression symptoms | 0.34 | 0.10 | 0.58 | 0.01 | Mental health domain |
| Increasing | Anxiety symptoms | 0.03 | -0.21 | 0.27 | 0.82 | Mental health domain |
| Decreasing | Anxiety symptoms | 0.16 | -0.08 | 0.41 | 0.19 | Mental health domain |
| Increasing | Inattentive and hyperactive symptoms | 0.62 | 0.38 | 0.86 | 0.00 | Mental health domain |
| Decreasing | Inattentive and hyperactive symptoms | 0.06 | -0.18 | 0.29 | 0.63 | Mental health domain |
| Increasing | Conduct disorder symptoms | 0.27 | 0.00 | 0.55 | 0.05 | Mental health domain |
| Decreasing | Conduct disorder symptoms | 0.22 | -0.04 | 0.48 | 0.10 | Mental health domain |
| Increasing | Alcohol dependence symptoms | -0.22 | -0.41 | -0.02 | 0.03 | Mental health domain |
| Decreasing | Alcohol dependence symptoms | 0.08 | -0.21 | 0.36 | 0.60 | Mental health domain |
| Increasing | Cannabis dependence symptoms | 0.11 | -0.14 | 0.37 | 0.39 | Mental health domain |
| Decreasing | Cannabis dependence symptoms | 0.43 | 0.03 | 0.82 | 0.03 | Mental health domain |
| Increasing | Experiences of psychosis | 0.22 | -0.05 | 0.49 | 0.11 | Mental health domain |
| Decreasing | Experiences of psychosis | 0.51 | 0.23 | 0.79 | 0.00 | Mental health domain |
| Increasing | BMI | 0.30 | 0.01 | 0.59 | 0.04 | Physical health domain |
| Decreasing | BMI | 0.07 | -0.15 | 0.28 | 0.55 | Physical health domain |
| Increasing | Log CRP | 0.03 | -0.24 | 0.30 | 0.82 | Physical health domain |
| Decreasing | Log CRP | 0.18 | -0.12 | 0.47 | 0.23 | Physical health domain |
| Increasing | Physical activity | -0.31 | -0.54 | -0.07 | 0.01 | Physical health domain |
| Decreasing | Physical activity | -0.53 | -0.73 | -0.33 | 0.00 | Physical health domain |
| Increasing | Daily number of cigarettes smoked | 0.38 | -0.04 | 0.80 | 0.08 | Physical health domain |
| Decreasing | Daily number of cigarettes smoked | 0.15 | -0.05 | 0.35 | 0.15 | Physical health domain |
| Increasing | Loneliness | 0.30 | 0.04 | 0.56 | 0.02 | Coping and functioning domain |
| Decreasing | Loneliness | 0.36 | 0.10 | 0.61 | 0.01 | Coping and functioning domain |
| Increasing | Life satisfaction | -0.32 | -0.58 | -0.07 | 0.01 | Coping and functioning domain |
| Decreasing | Life satisfaction | -0.34 | -0.59 | -0.08 | 0.01 | Coping and functioning domain |
| Increasing | Technology use | 0.27 | 0.02 | 0.53 | 0.03 | Coping and functioning domain |
| Decreasing | Technology use | 0.25 | 0.00 | 0.51 | 0.05 | Coping and functioning domain |
| Increasing | Coping with stress | -0.30 | -0.54 | -0.07 | 0.01 | Coping and functioning domain |
| Decreasing | Coping with stress | -0.19 | -0.41 | 0.03 | 0.08 | Coping and functioning domain |
| Increasing | PSQI score | 0.30 | 0.08 | 0.53 | 0.01 | Coping and functioning domain |
| Decreasing | PSQI score | 0.12 | -0.10 | 0.34 | 0.28 | Coping and functioning domain |
| Increasing | Job preparedness: skills | -0.05 | -0.32 | 0.21 | 0.69 | Employment domain |
| Decreasing | Job preparedness: skills | 0.03 | -0.21 | 0.27 | 0.80 | Employment domain |
| Increasing | Job preparedness: attributes | -0.39 | -0.67 | -0.10 | 0.01 | Employment domain |
| Decreasing | Job preparedness: attributes | -0.13 | -0.36 | 0.10 | 0.28 | Employment domain |
| Increasing | Job optimism | -0.56 | -0.81 | -0.32 | 0.00 | Employment domain |
| Decreasing | Job optimism | -0.39 | -0.62 | -0.16 | 0.00 | Employment domain |
| Increasing | Job search activities | -0.14 | -0.37 | 0.09 | 0.22 | Employment domain |
| Decreasing | Job search activities | 0.09 | -0.18 | 0.36 | 0.50 | Employment domain |

## Outcomes controlling for antecedents

### Logistic

| **Table 33.** Logistic outcome regressions: posterior probability sensitivity analysis (N=2141); controlling for antecedents | | | | | | |
| --- | --- | --- | --- | --- | --- | --- |
| **Class** | **Variable** | **OR** | **95% CI low** | **95% CI high** | **p value** | **Domain** |
| Increasing | Depression diagnosis | 1.05 | 0.57 | 1.92 | 0.88 | Mental health domain |
| Decreasing | Depression diagnosis | 1.41 | 0.78 | 2.57 | 0.26 | Mental health domain |
| Increasing | Anxiety diagnosis | 0.93 | 0.34 | 2.52 | 0.89 | Mental health domain |
| Decreasing | Anxiety diagnosis | 1.40 | 0.62 | 3.19 | 0.42 | Mental health domain |
| Increasing | ADHD diagnosis | 1.04 | 0.49 | 2.24 | 0.91 | Mental health domain |
| Decreasing | ADHD diagnosis | 0.73 | 0.32 | 1.69 | 0.46 | Mental health domain |
| Increasing | Moderate conduct disorder | 1.27 | 0.68 | 2.40 | 0.45 | Mental health domain |
| Decreasing | Moderate conduct disorder | 1.13 | 0.55 | 2.30 | 0.75 | Mental health domain |
| Increasing | Alcohol dependence | 0.38 | 0.13 | 1.08 | 0.07 | Mental health domain |
| Decreasing | Alcohol dependence | 0.94 | 0.44 | 1.99 | 0.86 | Mental health domain |
| Increasing | Cannabis dependence | 1.23 | 0.47 | 3.20 | 0.67 | Mental health domain |
| Decreasing | Cannabis dependence | 1.57 | 0.65 | 3.79 | 0.32 | Mental health domain |
| Increasing | PTSD lifetime diagnosis | 1.78 | 0.79 | 4.02 | 0.16 | Mental health domain |
| Decreasing | PTSD lifetime diagnosis | 1.49 | 0.60 | 3.71 | 0.39 | Mental health domain |
| Increasing | PTSD current diagnosis | 1.46 | 0.44 | 4.90 | 0.54 | Mental health domain |
| Decreasing | PTSD current diagnosis | 1.07 | 0.29 | 3.85 | 0.92 | Mental health domain |
| Increasing | Suicide attempt | 1.87 | 0.99 | 3.53 | 0.05 | Mental health domain |
| Decreasing | Suicide attempt | 1.90 | 0.99 | 3.64 | 0.05 | Mental health domain |
| Increasing | Service use | 1.53 | 0.77 | 3.07 | 0.23 | Mental health domain |
| Decreasing | Service use | 0.83 | 0.38 | 1.79 | 0.63 | Mental health domain |
| Increasing | Not in employment or education | 1.16 | 0.64 | 2.12 | 0.62 | Employment domain |
| Decreasing | Not in employment or education | 0.83 | 0.38 | 1.80 | 0.64 | Employment domain |

### Linear

| **Table 34.** Linear outcome regressions: posterior probability sensitivity analysis (N=2141); controlling for antecedents | | | | | | |
| --- | --- | --- | --- | --- | --- | --- |
| **Class** | **Variable** | **Beta estimate** | **95% CI low** | **95% CI high** | **p value** | **Domain** |
| Increasing | Depression symptoms | 0.11 | -0.16 | 0.38 | 0.42 | Mental health domain |
| Decreasing | Depression symptoms | 0.24 | -0.03 | 0.52 | 0.08 | Mental health domain |
| Increasing | Anxiety symptoms | -0.12 | -0.37 | 0.13 | 0.35 | Mental health domain |
| Decreasing | Anxiety symptoms | 0.01 | -0.27 | 0.30 | 0.92 | Mental health domain |
| Increasing | Inattentive and hyperactive symptoms | 0.36 | 0.10 | 0.62 | 0.01 | Mental health domain |
| Decreasing | Inattentive and hyperactive symptoms | -0.15 | -0.41 | 0.11 | 0.26 | Mental health domain |
| Increasing | Conduct disorder symptoms | 0.15 | -0.15 | 0.44 | 0.32 | Mental health domain |
| Decreasing | Conduct disorder symptoms | 0.08 | -0.20 | 0.36 | 0.59 | Mental health domain |
| Increasing | Alcohol dependence symptoms | -0.29 | -0.48 | -0.10 | 0.00 | Mental health domain |
| Decreasing | Alcohol dependence symptoms | 0.04 | -0.29 | 0.36 | 0.83 | Mental health domain |
| Increasing | Cannabis dependence symptoms | 0.04 | -0.25 | 0.32 | 0.80 | Mental health domain |
| Decreasing | Cannabis dependence symptoms | 0.29 | -0.11 | 0.69 | 0.15 | Mental health domain |
| Increasing | Experiences of psychosis | 0.02 | -0.26 | 0.30 | 0.89 | Mental health domain |
| Decreasing | Experiences of psychosis | 0.34 | 0.02 | 0.65 | 0.04 | Mental health domain |
| Increasing | BMI | 0.26 | -0.05 | 0.58 | 0.10 | Physical health domain |
| Decreasing | BMI | 0.08 | -0.18 | 0.33 | 0.56 | Physical health domain |
| Increasing | Log CRP | 0.05 | -0.24 | 0.33 | 0.74 | Physical health domain |
| Decreasing | Log CRP | 0.20 | -0.13 | 0.53 | 0.23 | Physical health domain |
| Increasing | Physical activity | -0.23 | -0.49 | 0.03 | 0.08 | Physical health domain |
| Decreasing | Physical activity | -0.36 | -0.60 | -0.13 | 0.00 | Physical health domain |
| Increasing | Daily number of cigarettes smoked | 0.30 | -0.14 | 0.74 | 0.18 | Physical health domain |
| Decreasing | Daily number of cigarettes smoked | -0.06 | -0.30 | 0.18 | 0.60 | Physical health domain |
| Increasing | Loneliness | 0.18 | -0.07 | 0.44 | 0.16 | Coping and functioning domain |
| Decreasing | Loneliness | 0.23 | -0.06 | 0.53 | 0.12 | Coping and functioning domain |
| Increasing | Life satisfaction | -0.19 | -0.46 | 0.08 | 0.16 | Coping and functioning domain |
| Decreasing | Life satisfaction | -0.18 | -0.48 | 0.11 | 0.21 | Coping and functioning domain |
| Increasing | Technology use | 0.12 | -0.15 | 0.38 | 0.39 | Coping and functioning domain |
| Decreasing | Technology use | 0.12 | -0.16 | 0.40 | 0.41 | Coping and functioning domain |
| Increasing | Coping with stress | -0.15 | -0.40 | 0.10 | 0.23 | Coping and functioning domain |
| Decreasing | Coping with stress | 0.02 | -0.23 | 0.27 | 0.86 | Coping and functioning domain |
| Increasing | PSQI score | 0.22 | -0.03 | 0.47 | 0.09 | Coping and functioning domain |
| Decreasing | PSQI score | 0.02 | -0.24 | 0.28 | 0.90 | Coping and functioning domain |
| Increasing | Job preparedness: skills | 0.06 | -0.22 | 0.34 | 0.67 | Employment domain |
| Decreasing | Job preparedness: skills | 0.24 | -0.03 | 0.51 | 0.08 | Employment domain |
| Increasing | Job preparedness: attributes | -0.19 | -0.50 | 0.11 | 0.22 | Employment domain |
| Decreasing | Job preparedness: attributes | 0.18 | -0.10 | 0.45 | 0.20 | Employment domain |
| Increasing | Job optimism | -0.35 | -0.61 | -0.09 | 0.01 | Employment domain |
| Decreasing | Job optimism | -0.11 | -0.37 | 0.14 | 0.39 | Employment domain |
| Increasing | Job search activities | -0.21 | -0.46 | 0.04 | 0.10 | Employment domain |
| Decreasing | Job search activities | 0.11 | -0.18 | 0.40 | 0.45 | Employment domain |
| **Note.** Significant results that did not appear in the original analysis are marked in red. | | | | | | |

# Appendix S12. 3-Step sensitivity analysis

#

## Antecedents: Manual 3-step approach for predictors in Mplus

We used the manual 3-step approach whereby we manually set the mean structure for each of the classes (using logit values in the output from the 3-trajectory model) when running the regression model. The model then recognises the class/trajectory membership variable as a latent class variable. The model simultaneously regresses the increasing class on all variables (in one regression) and then the same for the decreasing class. We selected the output containing odds ratios (OR) using class 1 (Low stable) as the reference class to be reported. Using this approach, missing data is excluded (Number of cases with missing on x-variables: 550). Sex was not controlled for here.

Results from the manual 3-step method replicated those using the hard class classification reported in the manuscript.

### Increasing class regressions (Increasing class ~ variable1 + variable2 …)

| **Table 35**. Regression estimates and standard errors for the manual 3-step approach using Mplus: Increasing class | | | |
| --- | --- | --- | --- |
|  | **Estimate** | **Standard error** | **p value** |
| SES | 0.886 | 0.247 | 0.643 |
| ACORN classification | 1.199 | 0.477 | 0.676 |
| Vandalism | 0.855 | 0.102 | 0.157 |
| Problems with neighbours | 1.139 | 0.144 | 0.335 |
| Number of children in school | 1 | 0.001 | 0.753 |
| Children eligible for free school meals | 1 | 0.015 | 0.978 |
| Child harm | 1.16 | 0.526 | 0.761 |
| Total number of siblings | 0.848 | 0.128 | 0.236 |
| Social support | 0.981 | 0.026 | 0.466 |
| Activities with mother | 0.867 | 0.076 | 0.081 |
| Mum not lived with biological father | 1.241 | 0.67 | 0.719 |
| Any domestic violence | 0.742 | 0.271 | 0.342 |
| Maternal warmth | 0.981 | 0.16 | 0.906 |
| Maternal depression | 1.093 | 0.425 | 0.826 |
| Maternal openness | 0.996 | 0.03 | 0.905 |
| Maternal conscientiousness | 0.994 | 0.034 | 0.871 |
| Maternal extroversion | 1.01 | 0.038 | 0.802 |
| Maternal agreeableness | 0.988 | 0.028 | 0.665 |
| Maternal neuroticism | 0.989 | 0.032 | 0.721 |
| Parental antisocial behaviour | 1 | 0.01 | 0.966 |
| Parental alcoholism | 0.967 | 0.022 | 0.128 |
| Child IQ | 1.003 | 0.012 | 0.772 |
| Child executive functioning | 1.098 | 0.052 | **0.058** |
| Child theory of mind | 0.936 | 0.045 | 0.154 |
| Child externalising behaviours | 1.02 | 0.014 | 0.162 |
| Child internalising behaviours | 1.056 | 0.035 | 0.109 |
| Child ADHD behaviours | 1.064 | 0.016 | **<0.001** |
| Child prosocial behaviours | 1 | 0.03 | 0.998 |
| *Note*. Significant associations are shown in **bold** | | | |

### Decreasing class regressions (Decreasing class ~ variable1 + variable2 …)

| **Table 36**. Regression estimates and standard errors for the manual 3-step approach using Mplus: Increasing class | | | |
| --- | --- | --- | --- |
|  | **Estimate** | **Standard error** | ***p* value** |
| SES | 1 | 0.391 | 1 |
| ACORN classification | 0.286 | 0.244 | 0.003 |
| Vandalism | 0.851 | 0.158 | 0.345 |
| Problems with neighbours | 0.907 | 0.206 | 0.651 |
| Number of children in school | 1.004 | 0.002 | 0.016 |
| Children eligible for free school meals | 1.029 | 0.024 | 0.222 |
| Child harm | 1.3 | 0.631 | 0.634 |
| Total number of siblings | 1.216 | 0.161 | 0.18 |
| Social support | 0.964 | 0.04 | 0.362 |
| Activities with mother | 1.019 | 0.105 | 0.856 |
| Mum not lived with biological father | 0.876 | 0.532 | 0.816 |
| Any domestic violence | 0.704 | 0.411 | 0.472 |
| Maternal warmth | 1.16 | 0.22 | 0.467 |
| Maternal depression | 1.187 | 0.548 | 0.733 |
| Maternal openness | 1.092 | 0.037 | 0.013 |
| Maternal conscientiousness | 0.981 | 0.03 | 0.517 |
| Maternal extroversion | 0.946 | 0.044 | 0.228 |
| Maternal agreeableness | 1.004 | 0.046 | 0.925 |
| Maternal neuroticism | 1.001 | 0.054 | 0.985 |
| Parental antisocial behaviour | 1.003 | 0.012 | 0.796 |
| Parental alcoholism | 1.008 | 0.036 | 0.816 |
| Child IQ | 0.986 | 0.016 | 0.385 |
| Child executive functioning | 0.886 | 0.046 | 0.012 |
| Child theory of mind | 1.02 | 0.075 | 0.786 |
| Child externalising behaviours | 1.023 | 0.02 | 0.249 |
| Child internalising behaviours | 1.389 | 0.092 | <0.001 |
| Child ADHD behaviours | 1.056 | 0.021 | 0.008 |
| Child prosocial behaviours | 0.908 | 0.033 | 0.006 |
| *Note.* Significant (p<0.05) associations are shown in bold | | | |

## Outcomes: Automatic 3-Step BCH method sensitivity analysis in Mplus

The BCH method was first described by (Vermunt, 2010) and recently refined by (Asparouhov & Muthen, 2021). The BCH method outperforms the typical auxiliary method as it avoids shifts in latent class in the final stage that can happen using the auxiliary 3-step method. The BCH method uses a weighted multiple group analysis, where the groups correspond to the pre-estimated known latent classes. BCH also performs well when the variance of the variables differ substantially across classes. However, this approach uses equality tests of means across classes, not regression. All outcome variables (both binary and continuous) were entered simultaneously.

Results from the BCH method replicate those using the hard class classification reported in the manuscript.

| **Table 37.** Automatic 3-step BCH mean difference test for outcomes: Low stable class used as comparison group. | | | |
| --- | --- | --- | --- |
| **Variable** | **Class comparison** | **Chi square** | ***p* value** |
| Depression diagnosis | Increasing | 1.236 | 0.266 |
| Anxiety diagnosis | Increasing | 0.925 | 0.336 |
| ADHD diagnosis | Increasing | 4.424 | **0.035** |
| Conduct disorder diagnosis | Increasing | 7.823 | **0.005** |
| Alcohol dependence | Increasing | 0.666 | 0.415 |
| Cannabis dependence | Increasing | 3.949 | **0.047** |
| PTSD diagnosis: lifetime | Increasing | 1.162 | 0.281 |
| PTSD diagnosis: Current | Increasing | 0.436 | 0.509 |
| Psychotic experiences | Increasing | 8.742 | **0.003** |
| Self harm or suicide | Increasing | 4.666 | **0.031** |
| Service use | Increasing | 3.433 | 0.064 |
| Not in employment or education | Increasing | 9.257 | **0.002** |
| Depression symptoms | Increasing | 2.696 | 0.101 |
| Anxiety symptoms | Increasing | 0.256 | 0.613 |
| ADHD symptoms | Increasing | 29.268 | **0.000** |
| Conduct disorder symptoms | Increasing | 12.461 | **0.000** |
| Alcohol dependence symptoms | Increasing | 0.670 | 0.413 |
| Cannabis dependence symptoms | Increasing | 3.623 | 0.057 |
| BMI | Increasing | 2.624 | 0.105 |
| Log CRP | Increasing | 0.188 | 0.665 |
| Physical activity | Increasing | 5.459 | **0.019** |
| Daily number of cigarettes smoked | Increasing | 7.143 | **0.008** |
| Loneliness | Increasing | 8.615 | **0.003** |
| Life satisfaction | Increasing | 9.274 | **0.002** |
| Technology use | Increasing | 5.280 | **0.022** |
| Coping with stress | Increasing | 12.503 | **0.000** |
| PSQI Score | Increasing | 5.573 | **0.018** |
| Job preparedness: Skills | Increasing | 0.005 | 0.942 |
| Job preparedness: Attributes | Increasing | 7.964 | **0.005** |
| Job optimism | Increasing | 33.637 | **0.000** |
| Job search activities | Increasing | 0.296 | 0.587 |
| Depression diagnosis | Decreasing | 3.020 | 0.082 |
| Anxiety diagnosis | Decreasing | 0.056 | 0.812 |
| ADHD diagnosis | Decreasing | 2.625 | 0.105 |
| Conduct disorder diagnosis | Decreasing | 1.349 | 0.245 |
| Alcohol dependence | Decreasing | 0.162 | 0.687 |
| Cannabis dependence | Decreasing | 3.112 | 0.078 |
| PTSD diagnosis: lifetime | Decreasing | 0.174 | 0.677 |
| PTSD diagnosis: Current | Decreasing | 0.047 | 0.828 |
| Psychotic experiences | Decreasing | 8.017 | **0.005** |
| Self harm or suicide | Decreasing | 2.964 | 0.085 |
| Service use | Decreasing | 0.000 | 0.985 |
| Not in employment or education | Decreasing | 3.585 | 0.058 |
| Depression symptoms | Decreasing | 5.340 | **0.021** |
| Anxiety symptoms | Decreasing | 0.131 | 0.717 |
| ADHD symptoms | Decreasing | 0.007 | 0.934 |
| Conduct disorder symptoms | Decreasing | 1.194 | 0.274 |
| Alcohol dependence symptoms | Decreasing | 0.103 | 0.749 |
| Cannabis dependence symptoms | Decreasing | 4.476 | **0.034** |
| BMI | Decreasing | 0.119 | 0.731 |
| Log CRP | Decreasing | 1.198 | 0.274 |
| Physical activity | Decreasing | 16.671 | **0.000** |
| Daily number of cigarettes smoked | Decreasing | 6.196 | **0.013** |
| Loneliness | Decreasing | 3.002 | 0.083 |
| Life satisfaction | Decreasing | 4.679 | **0.031** |
| Technology use | Decreasing | 2.797 | 0.094 |
| Coping with stress | Decreasing | 0.936 | 0.333 |
| PSQI Score | Decreasing | 0.391 | 0.532 |
| Job preparedness: Skills | Decreasing | 0.124 | 0.725 |
| Job preparedness: Attributes | Decreasing | 1.281 | 0.258 |
| Job optimism | Decreasing | 9.899 | **0.002** |
| Job search activities | Decreasing | 0.216 | 0.642 |
| *Note.* Significant (*p*<0.05) mean differences between the Low stable class and the comparison class are indicated in bold | | | |

# References

Achenbach, T. M., & Edelbrock, C. (1991). *Child behavior checklist* (Vol. 7, pp. 371–391). Burlington (Vt).

American Psychiatric Association. (1994). *Diagnostic and statistical manual of mental disorders, 4th edn*. Washington, DC: American Psychiatric Association.

Asparouhov, T., & Muthen, B. (2021). Auxiliary variables in mixture modeling: Using the BCH method in Mplus to estimate a distal outcome model and an arbitrary secondary model. *Mplus Web Notes: No. 21*, 80.

Benjamini, Y., & Hochberg, Y. (1995). Controlling the False Discovery Rate: A Practical and Powerful Approach to Multiple Testing. Journal of the Royal Statistical Society. Series B (Methodological), 57(1), 289–300.

Caspi, A., Moffitt, T. E., Morgan, J., Rutter, M., Taylor, A., Arseneault, L., Tully, L., Jacobs, C., Kim-Cohen, J., & Polo-Tomas, M. (2004). Maternal expressed emotion predicts children’s antisocial behavior problems: Using monozygotic-twin differences to identify environmental effects on behavioral development. *Developmental Psychology*, *40*(2), 149–161.

Gerstadt, C. L., Hong, Y. J., & Diamond, A. (1994). The relationship between cognition and action: Performance of children 312–7 years old on a stroop- like day-night test. *Cognition*, *53*(2), 129–153.

Grodzinsky, G. M., & Diamond, R. (1992). Frontal lobe functioning in boys with attention‐deficit hyperactivity disorder. *Developmental Neuropsychology*, *8*(4), 427–445.

Herle, M., Micali, N., Abdulkadir, M., Loos, R., Bryant-Waugh, R., Hübel, C., Bulik, C. M., & De Stavola, B. L. (2020). Identifying typical trajectories in longitudinal data: Modelling strategies and interpretations. *European Journal of Epidemiology*, *35*(3), 205–222.

Hughes, C., Adlam, A., Happé, F., Jackson, J., Taylor, A., & Caspi, A. (2000). Good test-retest reliability for standard and advanced false-belief tasks across a wide range of abilities. *Journal of Child Psychology and Psychiatry*, *41*(4), 483–490.

Jaffee, S. R., Caspi, A., Moffitt, T. E., & Taylor, A. (2004). Physical maltreatment victim to antisocial child: evidence of an environmentally mediated process. *Journal of Abnormal Psychology*, *113*(1), 44–55.

John, O. P. (1999). The big five trait taxonomy: History, measurement, and theoretical perspectives. In *Handbook of personality: Theory and research* (Vol. 2, pp. 102–138).

Odgers, C. L., Caspi, A., Bates, C. J., Sampson, R. J., & Moffitt, T. E. (2012). Systematic social observation of children’s neighborhoods using Google Street View: A reliable and cost-effective method: SSO in street view. *Journal of Child Psychology and Psychiatry*, *53*(10), 1009–1017.

Odgers, C. L., Caspi, A., Russell, M. A., Sampson, R. J., Arseneault, L., & Moffitt, T. E. (2012). Supportive parenting mediates neighborhood socioeconomic disparities in children’s antisocial behavior from ages 5 to 12. *Development and Psychopathology*, *24*(3), 705–721.

Pokorny, A. D., Miller, B. A., & Kaplan, H. B. (1972). The Brief MAST: A shortened version of the Michigan alcoholism screening test. *American Journal of Psychiatry*, *129*(3), 342–345.

Straus, M. A. (1979). Measuring intrafamily conflict and violence: The Conflict Tactics (CT) Scales. *Journal of Marriage and Family*, *41*(1), 75–88.

van der Nest, G., Lima Passos, V., Candel, M. J. J. M., & van Breukelen, G. J. P. (2020). An overview of mixture modelling for latent evolutions in longitudinal data: Modelling approaches, fit statistics and software. *Advances in Life Course Research*, *43*, 100323.

Vermunt, J. K. (2010). Latent class modeling with covariates: Two improved three-step approaches. *Political Analysis*, *18*(4), 450–469.

Wechsler, D. (1990). *Wechsler preschool and primary scale of intelligence – Revised.* London: The Psychological Corporation, Harcourt Brace Jovanovic.

Wickrama, K. K. A. S., Lee, T. K., O’Neal, C. W., & Lorenz, F. O. (2016). *Higher-order growth curves and mixture modeling with Mplus: A practical guide*. Routledge.
